# Peach Fruit Development: A Comparative Proteomic Study Between Endocarp and Mesocarp at Very Early Stages Underpins the Main Differential Biochemical Processes Between These Tissues

**DOI:** 10.3389/fpls.2019.00715

**Published:** 2019-06-04

**Authors:** Carlos E. Rodriguez, Claudia A. Bustamante, Claudio O. Budde, Gabriela L. Müller, María F. Drincovich, María V. Lara

**Affiliations:** ^1^Facultad de Ciencias Bioquímicas y Farmacéuticas, Centro de Estudios Fotosintéticos y Bioquímicos, Consejo Nacional de Investigaciones Científicas y Técnicas, Universidad Nacional de Rosario, Rosario, Argentina; ^2^Estación Experimental San Pedro, Instituto Nacional de Tecnología Agropecuaria, San Pedro, Argentina

**Keywords:** *Prunus persica*, fruit development, lignification, endocarp, mesocarp, asparagine, β–cyanoalanine hydratase, β–cyanoalanine synthase

## Abstract

Peach (*Prunus persica*) is an important economically temperate fruit. The development follows double sigmoid curve with four phases (S1–S4). We centered our work in the early development. In addition to S1, we studied the very early stage (E) characterized by the lag zone of the exponential growing phase S1, and the second stage (S2) when the pit starts hardening. “Dixiland” peach fruit were collected at 9 (E), 29 (S1), and 53 (S2) days after flowering (DAF) and endocarp and mesocarp were separated. There was a pronounced decrease in total protein content along development in both tissues. Quantitative proteomic allowed the identification of changes in protein profiles across development and revealed the main biochemical pathways sustaining tissue differentiation. Protein metabolism was the category most represented among differentially proteins in all tissues and stages. The decrease in protein synthesis machinery observed during development would be responsible of the protein fall, rather than a proteolytic process; and reduced protein synthesis during early development would reroute cell resources to lignin biosynthesis. These changes were accompanied by net decrease in total amino acids in E1–S1 and increase in S1–S2 transitions. Amino acid profiling, showed Asn parallels this trend. Concerted changes in Asn and in enzymes involved in its metabolism reveal that increased synthesis and decreased catabolism of Asn may conduct to an Asn increase during very early development and that the β-Cyano-Alanine synthase/β-Cyano-Alanine hydratase could be the pathway for Asn synthesis in “Dixiland” peach fruit. Additionally, photosynthetic machinery decays during early development in mesocarp and endocarp. Proteins related to photosynthesis are found to a higher extent in mesocarp than in endocarp. We conclude mesocarpic photosynthesis is possible to occur early on the development, first providing both carbon and reductive power and latter only reductive power. Together with proteomic, histological tests and anatomical analysis help to provide information about changes and differences in cells and cell-walls in both tissues. Collectively, this work represents the first approach in building protein databases during peach fruit development focusing on endocarp and mesocarp tissues and provides novel insights into the biology of peach fruit development preceding pit hardening.

## Introduction

Peach (*Prunus persica*) is a stone fruit of agricultural relevance, not only because of its economic value but also because of its relevance in human health as an important source of phenolic compounds, cyanogenic glucosides and phytoestrogens. In addition, peach has become the reference species for the Prunus family, which also encompasses other fruits such as berries, plums, apricots and almonds (Shulaev et al., 2008). Peach is a fleshy fruit consisting of a single seed surrounded by a pericarp. The pericarp is differentiated in three layers; the endocarp which is adjacent to the seed, the mesocarp consisting of the soft edible region of the fruit, and the exocarp or skin (Dardick and Callahan, 2014). Peach fruit is classified as a drupe, since during its development the endocarp undergoes a hardening process by secondary cell wall formation and lignin deposition.

Peach fruit development follows double sigmoid curve in which four phases can be defined (Tonutti et al., 1991), with growing occurring only during three of the stages and the interval corresponds to the stone formation (Callahan et al., 2009). The growth curve starts after pollination and fertilization. While the number of days of extension of each phase depends on the species, the typical features of each stage do not differ. The beginning is characterized by a rapid growth (exponential) and it is characterized by a high rate of cell division and elongation (S1). The extent of this phase is uniform along cultivars. During the second phase (S2), the endocarp starts becoming hardener to form the stone (Dardick et al., 2010). There is no net increase in fruit size at this stage and the duration is highly dependent on the cultivars, being shorter for early ripening varieties and longer for late ripening varieties (Bonghi et al., 2011). In the next step (S3), an exponential growth of the pericarp occurs again, which is the consequence of an increase in the cell division. In the last stage (S4), the fruit reaches its final size and ripening starts. S4 consist of S4-1, in which fruit gets its final size, and S4-2, when the fruit ripens in an ethylene dependent manner. S4-2 is the only phase that can take place even detached from the tree (Borsani et al., 2009).

The process of pit hardening has not been deeply studied (Dardick and Callahan, 2014). Early on, the presence of lignin in the stone was described by Ryugo (1961), as well as its biosynthetic intermediates (Ryugo, 1963). Later on, peroxidases and phenoloxidases were identified as enzymes involved in this process (Abeles and Biles, 1991; Alba et al., 1995, 2000). Hayama et al. (2006) identified cellulose synthase A1 as involved in cellulose synthesis in the endocarp during the hardening. Finally, Dardick et al. (2010) using the microarray technology, observed that certain genes linked to the phenylpropanoid pathway, lignin formation and flavonoid synthesis are transiently induced during lignification and subsequent stone hardening. They demonstrated that peach genes orthologous to *SHATTERPROOF, SEEDSTICK*, and *SECONDARY WALL THICKENING PROMOTING FACTOR 1* from *A. thaliana* are specifically expressed in the endocarp of the fruit, while the negative regulator *FRUITFUL* predominates in mesocarp and exocarp. They also revealed the coordination of the synthetic pathways of lignin and flavonoids during the early development of the fruit (Dardick et al., 2010). Later, Hu et al. (2011) showed that during development, while enzymes involved in lignin biosynthesis are up-regulated, enzymes like chalcone synthase, chalcone isomerase, anthocyanidin reductase, and leucoanthocyanidin dioxygenase, involved in the flavonoid pathways, are down-regulated in the endocarp at the beginning of S2.

Throughout the early stages of development, pericarp and seed/embryo are closely associated. When the pit is completely hard, this relationship becomes less strict (Ognjanov et al., 1995). Seed development and maturation has been earlier studied covering morphological aspects and biochemical (lipid and sugar contents) composition (Tukey, 1936; Ognjanov et al., 1995). More recently, Bonghi et al. (2011) performed transcriptomic analysis using seed and mesocarp from peach at S1 to S4 and identified marker genes for organ/tissue at each stage. Based on genes that respond to hormones, they proposed that auxin, cytokinins, and gibberellins are important signals for seed-mesocarp crosstalk during early development, while abscisic acid and ethylene act later.

In a previous work, by means of metabolomic studies and by analyzing the main regulatory enzymes of the identified metabolic processes, we analyzed the mesocarp pathways operating in the peach fruit mesocarp using “Dixiland” variety throughout development and maturation (Lombardo et al., 2011). At stage E, high levels of active polyphenols were detected, such as caffeoylquinic acids, which are substrates for the phenylpropanoid and lignin pathways during stone hardening. Sucrose levels showed a large increase during development (E1 to S4), mainly due to its translocation from the leaf. Interestingly, during early development, high levels of total proteins were observed in stage E, which decreased markedly in the mesocarp of S1 and S2. These results suggest that immature fruit store large amounts of protein, which could be later used to sustain the processes that are carried out in stages S1 and S2 (Lombardo et al., 2011). Therefore, the purpose of this research was to determine the reconfiguration of the proteome during the profound decrease in the protein levels that takes place at early stages of fruit development (from E to S2) comparing the endocarp and mesocarp separately in order to find out the main biochemical pathways that sustain the differentiation of these tissues.

## Materials and Methods

### Plant Material

*Prunus persica* (L.) Batsch cv “Dixiland” trees were grown at the Estación Experimental Agropecuaria INTA (33° 44′ 12.1″ south latitude and 59° 47′48.0″ west longitude). The orchard received routine horticultural care including winter and summer pruning, fruit thinning, fertilizing and pest control. Fruits were collected during the seasons 2015–2016, 2016–2017, and 2017–2018. Sampling was conducted as in Lombardo et al. (2011) as follows: 9 days after flowering DAF (E), 29 DAF (S1), and 53 DAF (S2).

Fresh fruit were manually pealed and dissected in mesocarp (m) and endocarp (e). Fresh material was used for histochemical procedures and weight measurements. The rest of the material was immediately frozen in liquid N_2_ and stored at -80°C for further experiments.

Dry (DW) and fresh (FW) weight were determined using at least ten fruits. For DW measurements, fruits were incubated at 80°C until constant weight.

### Free Amino Acid Analysis

The amino acid profile in peach fruit was assessed by Reverse Phase-HPLC and phenylisothiocyanate (PITC) derivatization as in Dhar et al. (2013). A C18 column (5 μm, 250 × 4.6 mm, LUNA Phenomenex) with a C18 guard security pre-column (4 × 3 mm) and an ÄKTA purifier equipment (GE Healthcare, Uppsala, Sweden) were used.

For amino acid extraction, 0.3 g of tissue were disaggregated in a mortar with 1 ml of 0.1 M HCl. After centrifugation at 14,000 g at 4°C, the supernatant was precipitated with 10% (v/v) TCA and maintained on ice during 30 min. After clarification, the amino acids were derivatized as follows. Fifty microliter of the supernatant were mixed with 50 μl of methanol/water/triethylamine (2:2:1, v/v) and dried immediately under vacuum. Then, PITC reagent (methanol/triethylamine/water/PITC, 7:1:1:1, v/v) was added and kept at room temperature for 20 min. After drying, the PITC derivatives were dissolved in 300 μl acetate buffer (mobile phase A).

HPLC was conducted as exactly described in Ruggieri et al. (2018). Mobile phase A consisted in sodium acetate trihydrate (pH 6.4) with 0.5 ml of triethylamine (TEA) and mobile phase B of acetonitrile: H_2_O (6: 4, v/v). All solutions were filtered through a 0.22 mm Millipore membrane. One hundred microliters of sample or standard were injected. Running conditions: a gradient between phases A and B was used (Supplementary Table 1A). The column was kept at 39°C and a flux of 1 ml/min. Amino acids were detected by measuring the absorbance at 254 nm.

Calibration curves were prepared by duplicate as reported in Ruggieri et al. (2018) using cysteine, arginine, histidine, isoleucine, leucine, lysine, methionine, phenylalanine, tyrosine, threonine, valine, alanine, aspartic acid, glutamic acid, glycine, proline, serine, asparagine, glutamine, cystine, ornithine, citrulline, and tryptophan as standards. Calibration equations are shown in Supplementary Table 1B. The amount of each amino acid in the samples was expressed as μmol per gram of fresh weight (μmol. gFW^-1^).

### RNA Extraction and cDNA Synthesis

RNA was extracted following the procedure described in Meisel et al. (2005) using 3 g of fresh tissue. Due to the small size of fruits at stage E, a pool of at least four fruits was used. Then total RNA was treated with DNase RQ1 (Promega). The quality of the extracted RNA was checked by electrophoresis and the concentration measured using the Take3^TM^ Micro-Volume Plate adaptor and a EPCOCH2 spectrophotometer (BioTek^R^). Three μg of RNA was retro-transcribed using oligo(dT) and Mo-MLV reverse transcriptase (Promega), according to the manufacturer’s instructions.

### Quantitative Real-Time PCR (qRT-PCR)

Quantitative real-time PCR was conducted in an Mx3005P QPCR (Agilent technologies, Stratagene) cycler equipped with MxPro QPCR version 4.10 software.

Reactions were performed in a final volume of 20 μl containing 1X Taq activity buffer (Promega), 200 μM dNTPs, 1 mM MgCl_2_, 0.8 U of GoTaq DNA polymerase enzyme (Promega), 0.5 μM of each primer, 0.5X SYBRGreen I (Invitrogen) and 1 μl of a fivefold dilution of each cDNA. Oligonucleotides primers were designed with the aid of Primer3 software^1^. Elongation factor 1 (ppa005702) was used as internal control (forward primer: 5′-TCCAGTTCTTGATTGCCACA-3′ and reverse primer 5′-CCATACCTGCATCTCCGTTC-3′). To amplify β–cyanoalanine hydratase (ppa008090) the following primers were used: 5′-CGCTGATTCCAGGGATGTAT-3′ (forward primer) and 5′-CCCATCATAATTGGGTCCAG-3′ (reverser primers).

The cycling parameters were as follows: an initial denaturation step at 94°C for 2 min; 40 cycles of 96°C for 10 s; 58°C for 15 s; 72°C for 1 min, and 77°C for 7 s to detect fluorescence, and final elongation step at 72°C for 4 min. Melting curves were generated by rising the temperature from 65 to 95°C. The resulting amplicons were separated in a 2% (w/v) agarose gel. Three biological and three technical replicates were conducted. Relative expression was estimated using the 2^-ΔΔCt^ method (Livak and Schmittgen, 2001).

### Histochemical Staining Procedures

Samples were taken from fruits, cut in cubes of 3–4 mm side and fixed at 4°C in 50% (v/v) ethanol, 10% formaldehyde and 5% (v/v) acetic acid for 2 days (the solution was renewed once). The samples were dehydrated with a graded ethanol series and embedded in paraffin. Cross sections, 8 μm thick, were made with a rotary microtome (E. Leitz Wetzlar, New York) and placed onto gelatine-coated slides for microscopy.

Sections were dewaxed and rehydrated with xylene and then ethanol series following standard protocols and used for the staining procedures as follows.

Samples were stained with 17.5 mg. ml^-1^ Calcofluor white (Sigma) for 5 min for visualization of cellulose. Sections were washed with 1X Phosphate-Buffered Saline (PBS) (pH 7.4) and mounted with anti-fade solution (0.1% (w/v) p-phenylenediamine and 50% (v/v) glycerol in 1X PBS).

Aniline blue was used to enhance the overall fluorescence of all plant cell walls (Smith and Mc Cully, 1978) and to follow modifications in the cell wall composition. The stained material was viewed with a microscope Nikon Eclipse TE-2000-E2 with confocal system Nikon C1Plus SiR using the following settings excitation = 405, 488, and 543 nm; emission = 450/435 nm (blue), 515/530 nm (green), and 605/675 nm (red). Images were acquired with the Nikon EZ-C1 software. For well width estimation, each image was then divided into nine square regions and five of them were analyzed. Three images were analyzed at each stage of development for each tissue. The following process was repeated until a stable value was found. Cell wall width was measured using the program “Image J”^2^ in sections at 90° with respect to the perimeter of the wall.

For polysaccharides, dewaxed sections were incubated in periodic acid (1% w/v) for 30 min, washed and then incubated with Schiff’s reagent (Biopur, Argentina) for 1 h. After rinsing, the sections were ready for observation with light microscopy. Images were acquired through a Nikon Labophot-2 Light microscope using a TV Lens C-0.45x Nikon digital camera Micrometrics SE (standard edition) Premium.

Lignin deposition was evidenced by the use of the Phloroglucinol staining using fresh fruit. A solution of 5% (w/v) phloroglucinol (Sigma) in 80% (v/v) methanol was applied to the fruit surface. After 5 min, some drops of HCL concentrated were added. The presence of lignified tissues was revealed as a red-violet coloration.

### Proteomic Analysis Using High–Resolution Mass Spectrometry

#### Protein Extraction

Total proteins were extracted from 0.5 g of fresh tissue using a buffer containing 50 mM Tris-HCl, pH 7; 1 mM EDTA; 0.5% (v/v) Triton X-100; 10 mM β-mercaptoethanol; 10% (p/v) glycerol; 2 mM MgCl_2_; 0.2 mM phenylmethylsulfonyl fluoride (PMSF) and polyvinyl polypirrolidone (PVPP).

#### Protein Quantitation

Protein concentration was determined according to Bradford (1976) using the Protein Assay reagent from Bio-Rad and BSA as standard.

#### Protein Modification and Proteomics

Forty micrograms of proteins extracted from mesocarp and endocarp of peaches at stages E, S1, and S2 were precipitated with 1/5 volumes of 100% (w/v) TCA overnight at -20°C. The pellet was washed twice with cold acetone and proteins were finally resuspended in 50 μl 8 M Urea and reduced with 10 mM DTT for 45 min at 56°C. After alkylation with 20 mM iodoacetamide for 40 min, proteins were precipitated with 1/5 100% (w/v) TCA overnight, washed with cold acetone, dried and delivered to the Proteomics Core Facility CEQUIBIEM, Buenos Aires, Argentina. Proteins were resuspended in 50 mM NH_4_HCO_3,_ pH 8 and digested overnight with sequencing-grade modified trypsin (Promega). Zip-Tip C18 (Merck Millipore) columns were used for desalting. Resulted peptides were separated in a nano-HPLC (EASY-nLC 1000, Thermo Fisher Scientific, Germany) coupled to a mass spectrometer with Orbitrap technology (Q-Exactive with High Collision Dissociation cell and Orbitrap analyzer, Thermo Fisher Scientific, Germany). Peptides were ionized by electrospray. Proteome Discoverer 2.1 software (ThermoScientific, Germany) and the peach reference proteome set from uniprot (*Prunus persica* (*Amygdalus persica*)-UP000006882-Uniprot) were used to identify peptides and proteins.

#### Differential Proteome Analysis

Statistical analysis of proteomics data was conducted using the Perseus software platform (Tyanova et al., 2016)^3^. Before analysis, data were normalized and subjected to manually missing-value imputation. Missing/zero values were replaced by the minimum value detected by the mass spectrometer (considered as the detection limit) when at least two of the three replicates were missing. Instead, when the peptide was detected in two of the three replicates the missing/zero values were left blank. LFQ protein intensities were log2 transformed.

Two-sample tests were conducted to compare proteomes of Ee vs. S1e; S1e vs. S2e; Em vs. S1m; S1m vs. S2m; Ee vs. Em; S1e vs. S1m; S2e vs. S2m; by applying the standard *t*-test statistic with a permutation-based false discovery rate of 0.05. A *q* ≤ 0.05 and a fold change (FC) < 0.5 or > 2 were used as significance threshold parameters. Three biological replicates were used for each sample analyzed (Ee, S1e, S2e, Em, S1m or S2m). The *p-*value was set at 0.05. Volcano plots showing *q-*values (-log2) were used to assess differences in Ee vs. S1e; S1e vs. S2e; Em vs. S1m; S1m vs. S2m; Ee vs. Em; S1e vs. S1m; S2e vs. S2m.

Ontology annotations of significantly regulated proteins for “cellular component,” “biological process,” and “molecular function” were analyzed to assess common localizations and functions by using MapMan (Usadel et al., 2009).

#### Data Statistical Analysis and Representation

With the exception of proteomic analysis where Perseus software was used and of cell width data, data was analyzed using one way-ANOVA. Minimum significance differences were calculated by the Bonferroni or Fisher tests (α = 0.05) using the Sigma Stat Package (Systat Software Inc., San Jose, CA, United States). The Kruskal-Wallis One Way Analysis of Variance on ranks followed by the non-parametric Dunn’s test (α = 0.05) was used for comparison of cell width measurements in each type of tissue and the Mann–Whitney *U*-test (α = 0.05) was used to compare the width of cell walls between endocarp and mesocarp, at each stage.

Principal component analysis (PCA) was conducted using the XLSTAT software (Microsoft Excel) and amino acid quantification data. In the case of proteins, PCA was conducted using Clustvis (Metsalu and Vilo, 2015).

For data visualization, MultiExperiment Viewer software was used (MeVv5.1.1, Saeed et al., 2003)^4^.

## Results

### “Dixiland” Peach Growing Curve

The first step in our analysis was the establishment of a growing curve in order to identify the different phases of the process. Supplementary Figure 1A shows the fresh weight of “Dixiland” peach fruit vs. the days after flowering (DAF). As shown in Lombardo et al. (2011) this cultivar exhibits the peach fruit typical growing curve. In addition to weight, volume, calculated using the formula for the volume of an elliptical spheroid, was used as indicative of fruit size. Supplementary Figure 1B shows the dramatic increase in fruit volume from E to S1 (42.3-fold) and a threefold thereafter.

**FIGURE 1:**
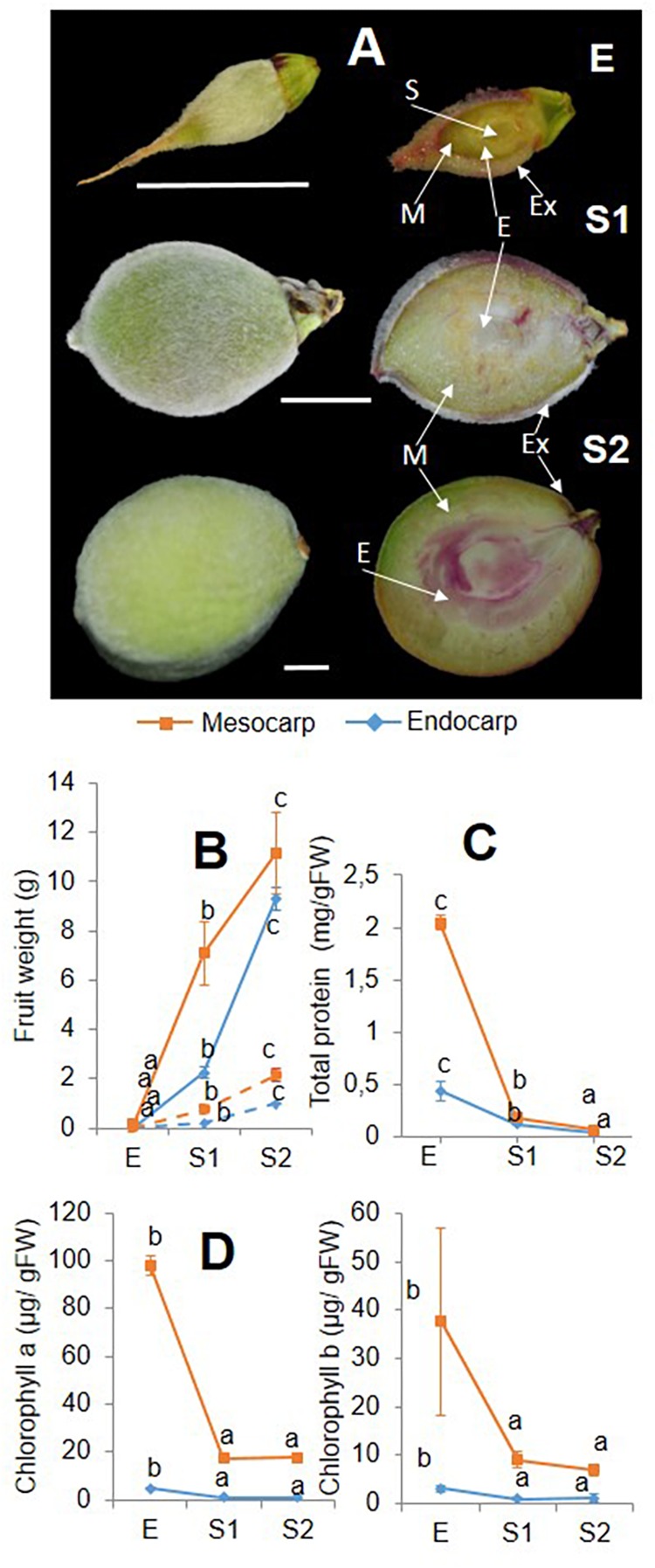
**(A)** Lignin staining during early developing of “Dixiland” peach fruit. Peach fruit were collected after 9 (E), 29 (S1) and 53 (S2) DAF and subjected to phloroglucinol–HCl staining to detect lignin deposition. Scale bars: 1 cm. S: seed; E: Endocarp; M: mesocarp; Ex: exocarp. Weight **(B)**, protein quantification **(C)** and chlorophyll analysis **(D)** were conducted in mesocarp (orange) and endocarp (blue) from peach fruit at E, S1, and S2 stages. Whole and dotted lines, respectively, represent fresh and dry weight curves. Total protein and chlorophylls are expressed in a fresh weight basis. Within each tissues, values with different letters are statically significant different (*p* < 0.05).

Lignin deposition, detected by phloroglucinol–HCl staining, was followed as a way of “stage check.” No coloration in the endocarp or the mesocarp was observed during E stage, and only positive reaction was observed in the exocarp (Figure 1A). During S1, in addition to exocarp, vascular bundles are stained in the endocarp and the mesocarp. In S2, lignin deposition is clearly observed in the endocarp as large regions of red coloration with a clear perimeter of lignin deposition surrounding the seed. In this stage, vascular bundles in the mesocarp are also stained.

Collected E, S1, and S2 fruit were dissected manually by separating the endocarp from the mesocarp and used for further analysis. Fresh and dry weight curves reveal the same trend of increase of weight evolution in each tissue (Figure 1B). Total protein content decreased from E to S2 in both tissues, with endocarp showing a reduction of 3.6-times from Ee to S1e and a similar trend of decrease from S1e to S2e. While a similar tendency was observed in the mesocarp during the S1m to S2m transition (2.9-fold), the decrease in total protein was more pronounced in the Em to S1m transition (11.4-fold) (Figure 1C). In addition, chlorophyll content was lower in endocarp with respect to the mesocarp. At all stages analyzed, chlorophyll a was around 20-fold higher in the mesocarp than in the endocarp and chlorophyll b was 10-times greater in mesocarp than the endocarp. Both chlorophylls tend to decrease from E to S2 in both tissues (Figure 1D).

### Fruit Proteomics During Early Development in Endocarp and Mesocarp

Quantitative proteomics was conducted using label-free based LC-MS in the endocarp and mesocarp of peach fruit at developmental stages E (Ee and Em), S1 (S1e and S1m), and S2 (S2e and S2m). The entire dataset of protein identification of each sample is presented in Supplementary Table 2. The amount of total identified proteins varied between tissues and stages. In endocarp, the number of different proteins was 654, 929, and 988, for Ee, S1e, and S2e, respectively. In contrast, the number of detected proteins in mesocarp tended to decrease from E to S2 (1154, 996, and 917, for Em, S1m, and S2m, respectively). Proteins detected ranged between 4 and 239.7 kDa and from 3.87 to 11.81 pI (Supplementary Table 2).

Principal component analysis (PCA) was conducted with all proteome data obtained (Supplementary Figure 2). First principal component (PC1) explained 31.1% of the variation, while the second (PC2) and the third (PC3) principal components accumulated a total variation of 53.2 and 73.1%, respectively. The plots show that the protein profiling at E, S1, and S2 in endocarp and mesocarp is unique for each tissue at each stage. The profiles of the mesocarp (Em, S1m, and S2m) are more closely related than those of endocarp (Ee, S1e, and S2e) throughout the period analyzed (Supplementary Figures 2B,C). On the other hand, proteomes of both tissues at S2 are the closest related since S2m and S2e group together in PC2 vs. PC1, PC3 vs. PC1, and PC3 vs. PC2 (Supplementary Figure 2). In the other stages (E and S1), the proteomes of mesocarp and endocarp seem to be more divergent (Supplementary Figure 2).

To identify the proteins with differential abundance (PDA) in the tissues and stages, the Perseus software platform was used. Comparisons were conducted by using *t*-test (*P* < 0.05, Student’s *t*-test). Increases in more than twofold and less than a half were considered of biological relevance. The results are first presented showing PDA along development in endocarp and mesocarp; and secondly, PDA between endocarp and mesocarp at each developmental stage.

An initial analysis was the evolution of the protein profile in endocarp and mesocarp during development. From 569 proteins statistically determined to be differentially abundant in S1e with respect to Ee, 457 increased in S1 with respect to E, and 112 decreased. Five hundred and one proteins differed in abundance in S2e with respect to S1e, of which 236 were increased in S2 with respect to S1, and 265 decreased. In mesocarp the number of differentially proteins was lower, with 423 proteins varying between Em and S1m, and 335 between S1m and S2m. While 177 proteins were increased in S1m with respect to Em, 171 proteins were increased in S2m with respect to S1m.

**FIGURE 2:**
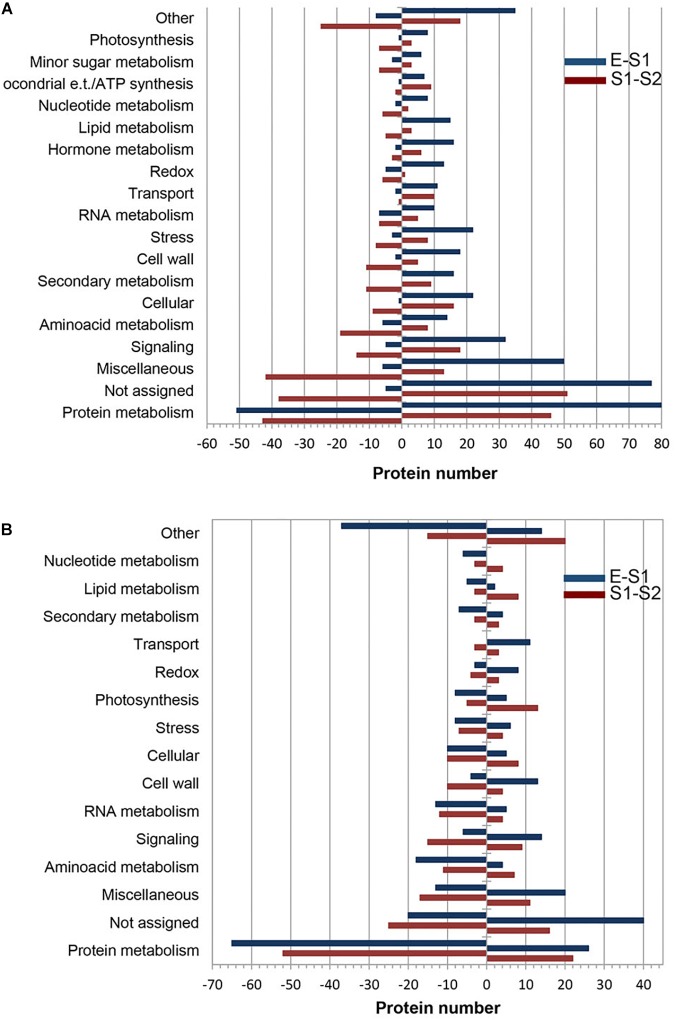
Functional classification of differentially expressed proteins over very early stages of peach fruit development. Proteins from endocarp **(A)** and mesocarp **(B)** tissues were analyzed at E, S1, and S2. Blue bars correspond to proteins increased (positive values) and decreased (negative values) in S1 with respect to E. Red bars represent the number of proteins increased (positive values) and decreased (negative values) in S2 with respect to S1.

With the aid of MapMan software (Supplementary Table 3) the distribution of PDA according to their functional category in endocarp (Figure 2A) and mesocarp (Figure 2B) was assessed. In both tissues and during the E to S1 and S1 to S2 transitions protein metabolism was the most represented functional category accounting between a 18 and 30% of all PDA. In all cases, not assigned and miscellaneous were the second and third over-represented categories. Amino acid metabolism, signaling, cell wall, cellular and stress were highly represented categories in the comparisons, as well. Particularly, during the transition from S1e to S2e, secondary metabolism represented a 4% of the total PDA. RNA metabolism represented the 5 and 6%, during the Em to S1m and S1m to S2m transitions, respectively. Notably, photosynthesis and lipid metabolism represented the 7 and 4% of proteins during S1m to S2m shift.

**FIGURE 3:**
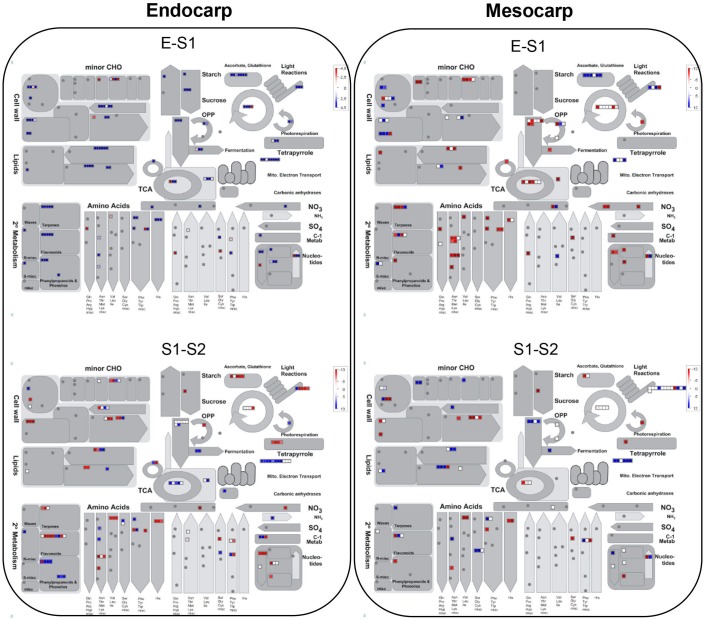
Overview of PDA in endocarp and mesocarp during the transitions from E to S1 and from S1 to S2 in relation to their correspondent metabolic pathways. Each square corresponds to a protein. Red and blue indicate lower and higher expression in the earlier stage of development, respectively, in a log2 basis. Scale bar is indicated at the top right of each figure. Images were generated using MapMan program (Usadel et al., 2009).

Figure 3 depicts an overview of the PDA involved in metabolic pathways modulated in mesocarp and endocarp during early “Dixiland” peach fruit development using MapMan program (Usadel et al., 2009). As it can be observed, during peach fruit development there is an important protein composition remodeling in both mesocarp and endocarp, with changes in the relative amount of proteins involved in cell wall, lipid, amino acid, carbohydrate, photosynthesis, energy, antioxidant, nucleotide, tetrapyrrole, N and S metabolisms (Figure 3).

**FIGURE 4:**
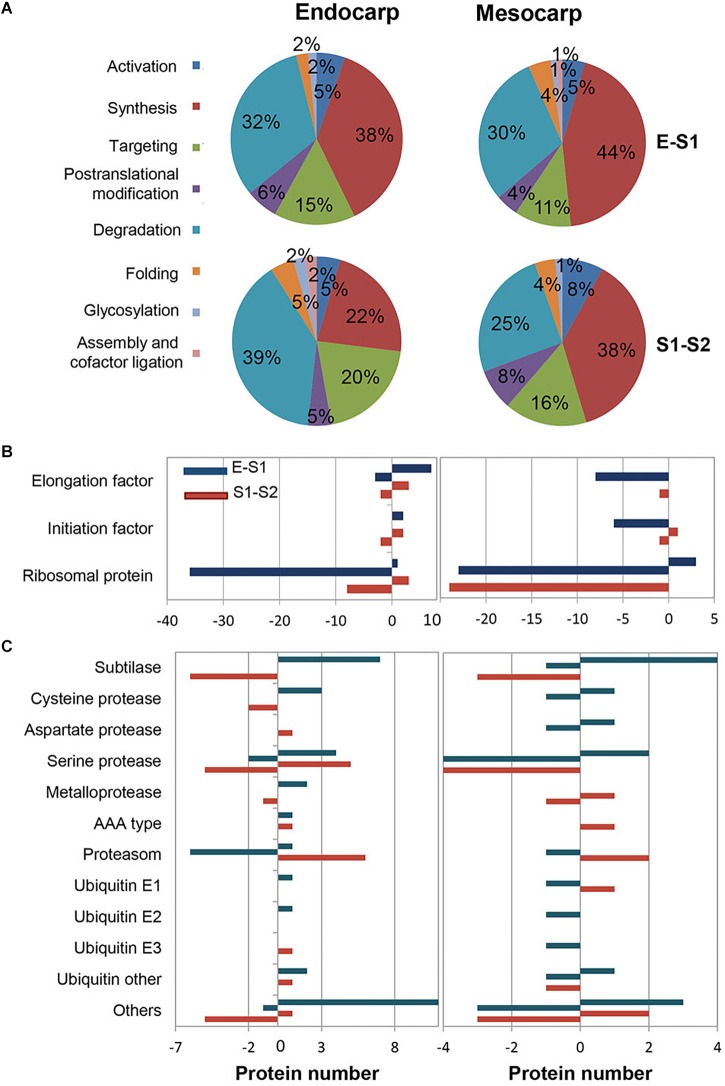
Distribution of variable proteins across very early stages of development within protein metabolism functional category. E1 to S1 and S1 to S2 transitions were analyzed in both endocarp and mesocarp. **(A)** Pie charts representing the total number of PDA distributed within “protein metabolism” GO terms subcategories. **(B)** Classification of PDA involved in protein biosynthesis in endocarp (left graph) and mesocarp (right graph). **(C)** Distribution of PDA participating in protein degradation in endocarp (left graph) and mesocarp (right graph). Blue bars correspond to proteins increased (positive values) and decreased (negative values) in S1 with respect to E. Red bars represent the number of proteins increased (positive values) and decreased (negative values) in S2 with respect to S1.

Considering that protein metabolism was the GO term most represented, the distribution of proteins within the subclasses was analyzed and shown in Figure 4. As expected, within all transitions protein synthesis and degradation were the most represented subgroups (Figure 4A). It is very interesting to note that within proteins involved in protein synthesis, those constituting the ribosome were the ones that varied the most, being the majority repressed in S1 with respect to E and in S2 with respect to S1 in both mesocarp and endocarp (Figure 4B). With respect to protein degradation, subtilases and serine proteases were the most represented, with subtilases being induced in the E to S1 transition and further repressed during S1–S2, in both endocarp and mesocarp (Figure 4C). While subunits of proteasome were repressed in E to S1 transition, they were induced in the transition from S1 to S2 (Figure 4C) in both endocarp and mesocarp. On the other hand, the different ubiquitins showed variable response in the different tissues and stages.

**FIGURE 5:**
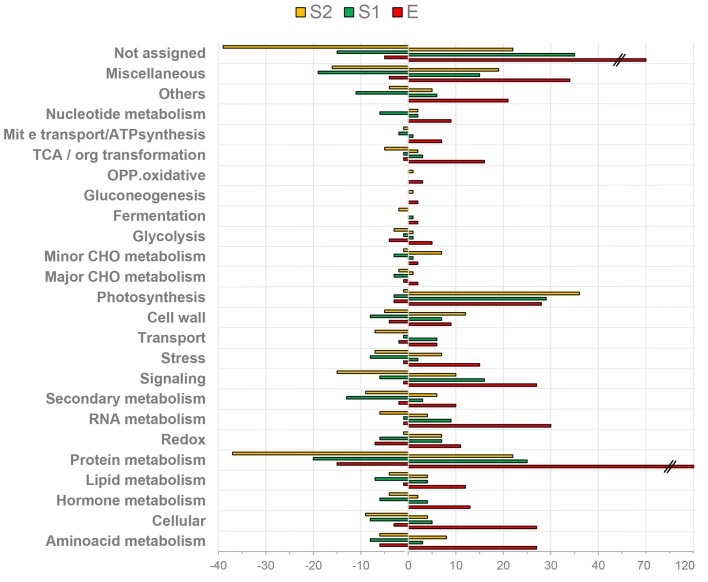
Functional classification of PDA in endocarp and in mesocarp. Positive and negative values represent the number of proteins increased and decreased, respectively, in mesocarp with respect to endocarp when proteomes of fruits at E (red bars), S1 (green bars) and S2 (yellow bars) were analyzed.

Further analysis focused on PDA between mesocarp and endocarp at each developmental stage. At stage E, 568 PDA were detected between endocarp and mesocarp. While 501 are increased in Em with respect to Ee, 61 were decreased. At S1, 341 proteins differed in their abundance, with 184 and 157 are increased and decreased, respectively, in mesocarp with respect to endocarp. Finally, 367 proteins vary in their amount between mesocarp and endocarp at S2. Of these, 163 are increased in mesocarp with respect of endocarp, and the rest are decreased. Figure 5 represents the distribution of PDA between mesocarp and endocarp according to the biological function. In general, a similar distribution not only with respect to the function but also to the proportion of increase and decrease is observed at stages S1 and S2. In contrast, at E a great number of proteins belonging to many functional categories (such as signaling, cellular, hormone, nucleotide, RNA, amino acid and lipid metabolism) are present in a greater extent in the mesocarp than in the endocarp. On the other hand, irrespectively of the stage of development, an overview of the PDA between tissues indicates that many proteins related to photosynthesis like those acting as structural components or binding chlorophyll in the photosystems, participating in the transport of electrons, in ATP synthesis, in the carbon reduction cycle and in photorespiration occur in a higher extent in the mesocarp with respect to the endocarp. A mean of 2000-fold of increase was detected for the different photosynthetic proteins. Supplementary Figure 3 shows the magnitude of variation of the photosynthetic-related proteins. In addition, enzymes involved in tetrapyrrole synthesis such as glutamate-1-semialdehyde 2,1-aminomutase (ppa005146m), porphobilinogen synthase (ppa006219m), protochlorophyllide reductase (ppa006788m), and magnesium chelatase (ppa006200m) were between 1000- and 6000-fold higher in the mesocarp than in the endocarp (Supplementary Tables 3E,F,G), in high agreement with chlorophyll measurements (Figure 1D). To clearly visualize the variable proteins between endocarp and mesocarp, at each stage, schemes representing metabolic pathways are shown in Supplementary Figure 4.

### Amino Acid Profiling at Early Peach Development

Considering that PDA involved in protein metabolism was the most represented category in endocarp and mesocarp during fruit development, amino acid profiling, conducted by phenylisothiocyanate (PITC) derivatization followed by HPLC, was analyzed in these tissues over fruit development. Not only relative amounts of each amino acid were revealed by this approach but also their absolute amounts due to the aid of calibrations curves. Amounts of each amino acid identified are shown in Supplementary Table 4 and expressed in μg per gram of fresh tissue. PCA of the data reveals that three PC explain the 84.7% of the variation (Supplementary Figure 5). The first PC explains a 40.2% of the variation, the second one the 29.4% and the third one the 15.1%. As it is the case of the proteome analysis, amino acid profiling of S2m and S2e group together in PC2 vs. PC1 and are closely related to S1e and S1m. In addition, it is clearly visualized that the profiles of E (either Ee or Em) appear in the plots distant from the other samples and of each other.

**FIGURE 6:**
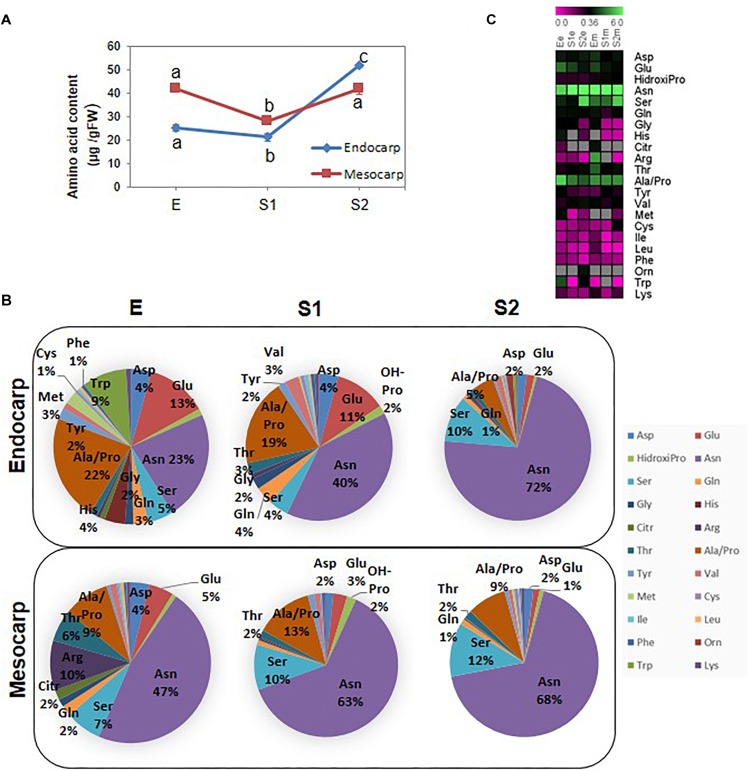
Amino acid profiling during early peach fruit development. **(A)** Total amino acid quantification in endocarp and mesocarp. Values represent the mean of six independent determinations. Error bars represent the standard deviation. Bars with at least one same letter are not statistically different within the same tissue (*p <* 0.001). **(B)** Pie charts showing the proportion of each amino acid in the endocarp and the mesocarp at the developmental stages E, S1, and S2. **(C)** Heat map showing the amount of each amino acid during development in the endocarp (e) and the mesocarp (m). The scale bar at the top of the figure represents the amount of each amino acid expressed in μg/GFW. Gray boxes indicate that the amino acid was not detected.

In order to have a clear picture of the relevance of the changes in amino acid composition, the total amount of free amino acids was calculated (Figure 6A). In both tissues, the total amount decrease from E to S1 and increase thereafter restoring the initial levels in mesocarp and exceeding the amounts at E in endocarp.

**FIGURE 7:**
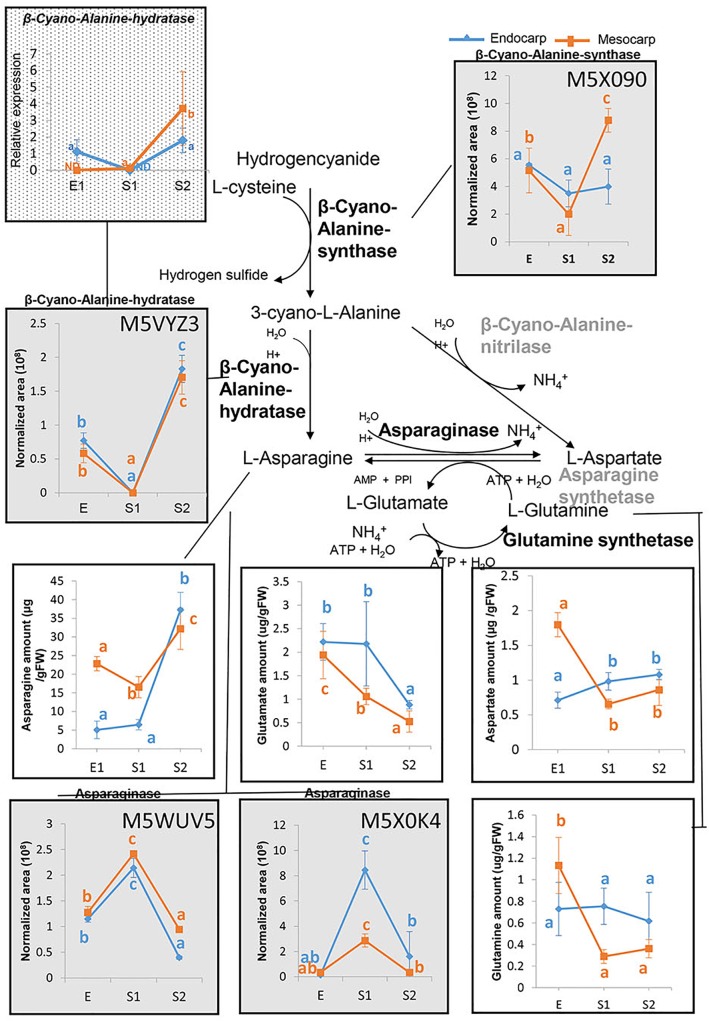
Asparagine metabolism in peach fruit. An overview of the metabolic pathways conducting to asparagine biosynthesis and metabolism is shown. Graphs in gray background represent the proteins profiles as assessed by nanoHPLC-MS (within each box the uniprot accession number of the protein is presented), graphs with a white background show the amounts of amino acids determined by PICT-HPLC and the graph with dotted background displays the relative expression of the transcript encoding β–Cyano-Alanine-hydratase analyzed by qRT-PCR. For each parameter and tissue, values with at least one same letter are not statistically different. Error bars represent the standard deviation. Enzymes in gray were not detected in the proteome of “Dixiland” peach fruit during the very early development. ND, not detected.

Figure 6B represents the percentage of each amino acid in a weight basis in mesocarp and endocarp at each developmental stage. The amount of each amino acid at the different developmental stages in endocarp and mesocarp is shown as a heat map (Figure 6C). Asparagine not only is the most abundant amino acid in the fruit under study but also it increases over development (Figure 7). On the other hand, other key amino acids involved in N metabolism, such as Gln, Asp, and Glu show a decline in mesocarp and endocarp. Serine, which is another abundant amino acid of the fruit, also tend to increase from E to S1 in both tissues. Neither the precursor in phenylpropanoid metabolism Phe nor its closely related amino acid Tyr exhibited changes in their amounts during development in mesocarp (Supplementary Table 4). Interestingly, Tyr increased and Phe decreased in S2 in endocarp.

Taking into account the highly abundance of Asn in peach fruit, the metabolic pathways in which it is involved were explored. For this purpose, the genes encoding the enzymes catalyzing its synthesis and degradation were explored in the peach genome^5^ based on known pathways. Once identified, the corresponding proteins were identified based on their uniprot accession number (Supplementary Table 5). These numbers were used to search the presence of these proteins in peach fruit proteome over development (Supplementary Table 2). To our surprise, none of the Asparagine synthetases identified in peach genome were found in peach proteome during very early development. In contrast, two Asparaginases (M5WUV5 and M5X0K4) were detected in both mesocarp and endocarp (Figure 7 and Supplementary Tables 2, 3). Both isoforms exhibited a similar trend of variation during the early development, which is opposite to that of the Asn profile. Moreover, neither Asp, nor Glu or Gln followed the trend of Asn variation during development.

Conversely, β–cyanoalanine synthase (β–CAS) and β–cyanoalanine hydratase producing L-Ans from L-Cys and hydrogencyanide were detected (Figure 7). The corresponding protein profiles are shown in Figure 7. In addition, as means of validation of these results, the transcript profile of β–CAS was also explored by qRT-PCR. Transcript profile agrees with that of the protein (Figure 7). In contrast, β–cyanoalanine nitrilase was not found in the proteome (Supplementary Table 2).

### Microscopic Confocal Analysis of Mesocarpic and Endocarpic Cells and Cell Walls During Development

A combination of histological tests, anatomical analysis and the use of confocal microscopy was used to provide information about the cell sizes and the cell walls of the endocarp and mesocarp during early development.

**FIGURE 8:**
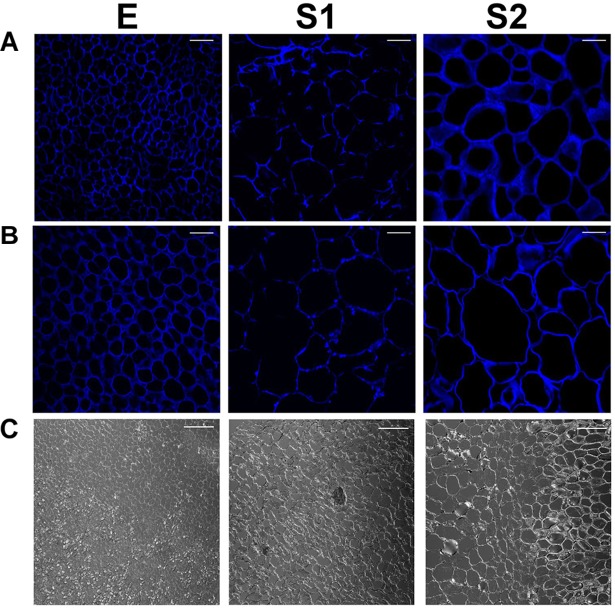
Calcofluor white staining of endocarpic **(A)** and mesocarpic **(B)** cells at E, S1, and S2. Magnification used: 60X. Scale bars: 25 μm. Bright field images of the interphases between endocarp and mesocarp **(C)**. Magnification used: 20X.

Figure 8 shows that at each stage analyzed, the sizes of the cells from the endocarpic tissue (Figure 8A) are always smaller than those of the mesocarp (Figure 8B). Bright field images on transition zone between endocarp and mesocarp allow the visualization of the different cells (Figure 8C). In addition, the size of both endocarpic and mesocarpic cells increases from E to S1, in agreement with increase in fruit size at this stage. Only a slight increase in cell dimension is observed in the transition from S1 to S2. To provide quantitative data on cell sizes, the number of cells per images of 51,042 μm^2^ collected with 60X magnification was counted at each stage and tissue and shown in Supplementary Figure 6. The number of cells per field at each stage is always smaller in the mesocarp than in the endocarp, denoting bigger sizes for cells in the mesocarp. Moreover, in each tissue, the number of cells per area is higher at E than at S1. There are no statically significant differences between measurements at S1 and S2 within each tissue (Supplementary Figure 6). Moreover, it is clearly observed the lower degree of calcofluor fluorescence, used to reveal cellulose, in both endocarp and mesocarp of fruit at S1 (Figure 8A,B). Therefore, lower amounts of cellulose are deposited in the cell walls at S1.

Confocal laser scanning microscopy images of aniline blue-stained sections (Supplementary Figure 7) show clear differences between endocarp and mesocarp. In addition, it is particularly notorious, the higher red fluorescence in the endocarp at S2 with respect to S1, in agreement with lignin staining (Figure 1A). Therefore, the staining was useful to reveal the differences in cell wall composition along fruit development in both endocarp and mesocarp. As control, autofluorescence was recorded in the absence of aniline blue to reveal, by comparing with stained images, the enhancement of endogenous fluorescence by the fluorochrome (Supplementary Figure 8).

**FIGURE 9:**
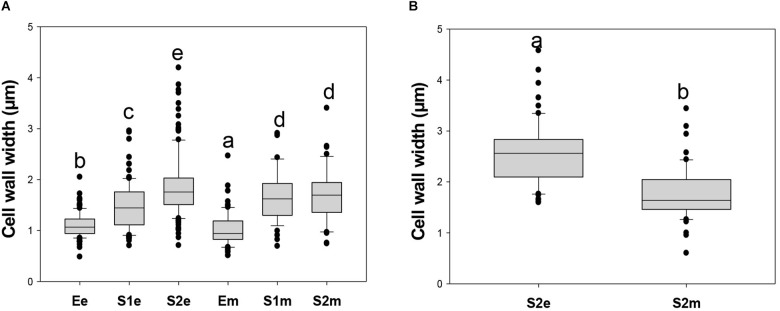
Box plots of cell wall thickness of endocarpic (e) and mesocarpic (m) cell walls in sections of fruit at E, S1, and S2 stages. **(A)** Cell wall width was measured using confocal laser scanning microscopy images of aniline blue-stained sections collected in the blue channel. Cell wall width was measured using the program “Image J” (http://imagej.nih.gov/ij) in sections at 90° with respect to the perimeter of the wall. Plots were constructed with Sigma Plot Software. Since the distribution of the data is not parametric, the Kruskal-Wallis One Way Analysis of Variance on ranks was applied followed by the Dunn’s Method for All Pairwise Multiple Comparison Procedure. **(B)** Cell wall width measured using images collected in the red channel from the endocarp and mesocarp of fruits collected at S2. Cell walls from S2e are thicker than from S2m (*p* < 0.001, Mann–Whitney *t*-test). Boxes with different letters are statistically different.

In addition, the single wall thickness of endocarpic and mesocarpic cell walls in sections of fruit at E, S1, and S2 stages were measured using the blue channel of images shown in Supplementary Figure 7 (Figure 9A). In the endocarp, cell wall width was increased from stage E to S2. On the other hand, in mesocarp, the thickness of the wall was increased in S1 with respect to E, and remained constant at S2. Moreover, wall width was always different for cells located at the endocarp, with respect to those at the mesocarp at stages E (*p* < 0.005) and S1 (*p* < 0.034). Since cell walls in the endocarp start becoming lignified at S2, images from the red channel were also used to measure the wall thickness. In this way, cell walls were found to be statically significant thicker (*p* < 0.001) in the endocarp than in the mesocarp at S2 (Figure 9B).

To aid in the analysis of cell walls during early development, changes in abundance of proteins related to the cell wall were investigated within data on proteomic analysis (Supplementary Table 3) and shown in Table 1. In general, and in agreement with an expansion in cell during the transition from E to S1, the enzymes involved in the synthesis of wall precursors are highly up-regulated in endocarp. Moreover, an upregulation of enzymes involved in cell wall modification (expansin, xyloglucan endotransglycosilases, and endoxyloglucan transferase), remodelation (pectinesterases), and degradation, as well as the incorporation of proteins targeted to the cell wall (fasciclin-like arabinogalactans and leucine-rich repeat family proteins/extensins) is observed in the endocarp and in the mesocarp. In contrast, when comparing S2 with respect to S1 a decrease in proteins under the mentioned categories is observed, denoting a cease in cell wall expansion at stage 2 (Table 1).

**Table 1:** Changes in proteins abundances involved in cell wall metabolism during very early devolvement of peach fruit.

Acc. No.	Protein description	Fold change (log2)			
		
		Endocarp		Mesocarp	
			
		E–S1	S1–S2	E–S1	S1–S2
**Cell wall precursor synthesis**					
ppa004903m	UDP-N-acetylglucosamine pyrophosphorylase-related AT1G31070	8.53			11.07
ppa004485m	ADP-glucose pyrophosphorylase family protein AT1G74910	11.65			
ppa007618m	Mannose-1-phosphate guanylyltransferaseAT2G39770	10.98			
ppa008032m	UDP-D-glucose/UDP-D-galactose 4-epimerase 5 AT4G10960		-10.50		
ppa006917m	UDP-glucuronate decarboxylase AT1G08200	1.05			
ppa005045m	UDP-glucose 6-dehydrogenase AT5G15490		-1.33		
ppa004626m	UDP-glucuronate decarboxylase 1 AT3G62830	8.33	4.10		
ppa005905m	UDP-GLUCURONIC ACID DECARBOXYLASE 1 AT3G53520		11.00		
ppa007623m	GHMP kinase family protein AT3G01640	10.70			
**Cell wall proteins: AGP, LRR and RGP**					
ppa007675m	Fasciclin-like arabinogalactan 2 AT4G12730	12.40		10.83	1.05
ppa023463m	Fasciclin-like arabinogalactan protein 8 precursor AT3G46550	11.40		8.34	4.18
ppa006726m	Fasciclin-like arabinogalactan protein 8 precursor AT5G55730	-1.91			
ppa010321m	Fasciclin-like arabinogalactan protein 7 precursor AT2G04780	11.22			
ppa023379m	Fasciclin-like arabinogalactan protein 7 precursor AT5G60490		12.23		
ppa006630m	Leucine-rich repeat family protein/extensin family protein AT4G29240	11.08		2.10	
ppa024854m	Leucine-rich repeat family protein/extensin family protein AT3G22800		-11.14	1.55	-12.53
ppa000722m	Leucine-rich repeat family protein/extensin family protein AT2G15880			-10.30	
ppa004109m	Leucine-rich repeat family protein AT1G49750			10.95	
ppa023082m	Leucine-rich repeat family protein/Receptor-like protein kinase AT4G06744				11.69
ppa014775m	Leucine-rich repeat family protein Receptor-like protein kinase AT1G49750				-12.23
ppa007588m	Alpha-1,4-glucan-protein synthase AT3G08900		1.23		
ppa007760m	Alpha-1,4-glucan-protein synthase AT3G08900			9.92	
**Cell wall degradation**					
ppa002559m	Glycosyl hydrolase family 3 protein AT5G04885	-3.12		1.17	
ppa015037m	Beta-D-xylosidase AT3G19620	12.51		12.83	
ppa005849m	(1-4)-beta-mannan endohydrolase AT5G01930	12.50	-12.50		
ppa001692m	Xylan 1,4-beta-xylosidase AT5G64570		-1.37		
ppa001675m	Beta-D-xylosidase AT1G78060		-11.70		-9.38
ppa001583m	Beta-D-xylosidase AT5G10560			-1.67	
ppa004996m	Polygalacturonase AT4G23500		-10.66		
ppa005599m	Polygalacturonase (pectinase) AT5G49215		-11.93		
ppa004793m	Polygalacturonase (pectinase) AT1G19170		11.88		
ppa005960m	Polygalacturonase (pectinase) AT3G57790				-2.16
ppa018224m	Polygalacturonase (pectinase) AT3G6149				-10.23
ppa005535m	Protein dehydration-induced protein RD22-like protein 2 AT5G25610				-12.37
ppa004101m	Protein dehydration-induced protein RD22-like protein 2 AT5G25610				-12.15
**Cell wall modification**					
ppa010171m	Expansin-like A1 precursor AT3G45970	10.49		11.25	-11.25
ppa009472m	Xyloglucan endotransglycosylase AT5G65730	10.52		10.97	
ppa009610m	Xyloglucan endotransglucosylase 6 AT4G25810			9.63	
ppa009387m	Endoxyloglucan transferase A4 AT5G13870			-10.93	
**Cell wall pectin esterases**					
ppa003697m	Pectinesterase-3 precursor AT1G11580	12.87	-12.87		
ppa004300m	Pectinesterase PPE8B precursor AT4G33220			-1.42	
ppa003307m	Pectinesterase 1 AT1G53840	12.13	-12.13		
ppa006668m	Pectinacetylesterase AT4G19420	12.54	-12.86	10.32	-10.32
ppa006718m	Pectinacetylesterase AT4G19420	1.58		1.24	-1.59


*The fold change in a log2 is presented. A positive FC in E–S1 and S1–S2 comparisons indicates an increase in the amount of protein in S1 and S2, respectively. Conversely, a negative value indicates a decrease. Prunus persica database (Phytozome) accession numbers are indicated. In addition, the Arabidopsis thaliana accession number is also provided with protein description. A blank indicates that there is no change registered in the protein in a specified comparison (the protein may remained unchanged or it could be undetected in both of the stages that are compared).*

Finally, PAS staining was conducted to reveal the presence of starch granules in plastids over development. While abundant starch grains were observed in mesocarp at E and S1, barely a few granules were observed at S2 (Supplementary Figures 9C,D). In contrast, granules were not observed in endocarp at S1 and S2 (Supplementary Figures 9A,B) but they were observed in E.

## Discussion

### Overall Endocarp and Mesocarp Proteome Reconfiguration During Peach Fruit Development

Peach fruit development shows a double sigmoidal curve (Supplementary Figure 1, Tonutti et al., 1997), with four phases (S1–S4). The first stage (S1) is characterized by an exponential growth of the fruit, as accounted by increases in fruit size and weight (Supplementary Figures 1A,B and Figure 1B) and lasting until 45 DAF. This phase has an initial lag period, which is named here as E. Results obtained show significant differences between E and S1, which include chlorophyll levels (Figure 1), protein content and profiling (Figure 1, 2, 4, 7), amino acid content and profiling (Figure 6) and starch content (Supplementary Figure 9); and thus, confirm the importance of the fractionated exploration of the E period. Another particular feature that our work includes is the dissection of the endocarp from the mesocarp during very early fruit development. In this sense, this proteomic approach reveals the uniqueness of the proteome of peach fruit at a stage very early after pollination (stage E), especially that of the endocarp (Ee), distantly in PCA plots from the proteomes of other stages and tissues (Supplementary Figure 2) and with numerous proteins occurring in a minor magnitude than in Em (Figure 5).

In addition, the proteome of the endocarp undergoes more pronounced remodeling over the development than that of the mesocarp; as it is shown by the closer association of mesocarpic samples (Em, S1m, and S2m) than the endocarpic profiles (Ee, S1e, and S2e) (Supplementary Figure 2B). Therefore, besides the mesocarp exhibits a decrease in total proteins of higher magnitude than that of the endocarp over early development, the changes in the protein profiling are less severe (Figure 1C). Moreover, despite the decrease in the net amount of proteins, the number of individually proteins identified tended to increase in the endocarp revealing changes in both protein quantity and quality (Supplementary Table 2). Previous high-throughput transcriptomic studies identified main genes and signaling pathways that regulate endocarp and mesocarp differentiation (Dardick and Callahan, 2014). Nevertheless, these studies are not enough to predict the resultant protein occurrences, since protein levels are also controlled by other mechanisms in addition to transcript levels. Thus, the work presented here represents the first approach in building protein databases during peach fruit development focusing on endocarp and mesocarp tissues.

### A Decrease in the Protein Synthesis Machinery During Early Development Conducts to a Fall in the Protein Content

In agreement with previous works the presence of lignin is observed in endocarp at S2 (Figure 1C; Dardick et al., 2010). This process is accompanied by an important number of PDA involved in secondary metabolism being present in the endocarp during the transition from S1 to S2 (Figure 2B and Supplementary Table 3) and in cellulose deposition (Figure 8). Lignin synthesis is a costly process, which has a great demand on reductive power and hydroxycinnamyl alcohols (or monolignols). Monolignols derive from the phenylpropanoid pathway that uses Phe as substrate (Vanholme et al., 2010). Thus, during very early peach development not only growth but also stone formation have a great demand on substrates. In the past few years, significant progress has been made in understanding seed development (Bonghi et al., 2011), stone formation (Dardick et al., 2010; Hu et al., 2011) and the pericarp growth (Bonghi et al., 2011; Lombardo et al., 2011). Proteins initially accumulated in peach have been proposed as a resource for lignification (Lombardo et al., 2011). Here, we have shown that the great decrease in protein content occurs not only in the endocarp but also in the mesocarp, with a different extent of variation (Figure 1C).

To get insight into the nature of total protein change over development (Figure 1C) we conducted quantitative proteomics. Protein metabolism was the category most represented among PDA in all tissues and stages analyzed (Figure 2). A repression in the ribosomal proteins reveals that a decrease in protein synthesis is a key component in the fall of total proteins during early development (Figure 4B), especially in the mesocarp during the Em to S1m transition (Figure 1C), where there is also a decrease in initiation and elongation factors involved in protein synthesis. Thus, given the contribution of ribosomes to cell weight, the decrease in the ribosomal proteins *per se* may significant contribute to the net drop in total proteins during the transition from E to S1. Besides, at this stage there is a reduction in the components of the ubiquitin-proteasome system indicating that proteins would not increase their decay through this pathway and would instead occur through other proteases (Figure 4C). In relation, the amount of amino acids does not increase in the E to S1 transition; suggesting that the protein mobilization to render amino acids as source of respiratory substrates (Araújo et al., 2011) is unlikely here. Considering that lignin synthesis starts very early during peach fruit development (Dardick et al., 2010; Figure 1A), it seems that protein synthesis would be reduced at this stage with the aim to reroute the resources to lignin biosynthesis. Similarly, in Arabidopsis the demands on basic metabolism and energy of the protein synthesis and degradation directly influences the cell growth (Piques et al., 2009).

Protein homeostasis depends on process of protein synthesis and degradation; and protein degradation plays a key role in plant growth, development and death (Palma et al., 2002). In contrast, our results show that in peach fruit during very early development the protein turnover could instead be more exerted at protein synthesis level. In this sense, ribosome modifications were found during development of Arabidopsis and bean leaves (Makrides and Goldthwaite, 1981; Schippers and Mueller-Roeber, 2010).

Following the E stage, in the S1–S2 transition, the system of ubiquitin-proteosome seems to be activated, as accounted by increases in the abundance of members of the proteasome 26S and ubiquitination process (Figure 4C) and in the increase in the content of total amino acids (Figure 6A). Nevertheless, the amino acid profiling shows that in each tissue the amino acid distribution at S1 and S2 are quite similar (Figure 6A,B), with the net increase in amino acid content mainly at expenses of increases in Asn (Figure 6C), ruling out the hypothesis of an increase of amino acids due to massive proteolysis; and, instead, more linked to Asn metabolism. In this sense, the occurrence and amount of enzymes involved in Asn metabolism reveals that the level of this amino acid, on one hand, is in parallel with the amount of β-CAS and β-cyanoalanine hydratase involved in Asn biosynthesis (Figure 7). On the other hand, the relative amounts of two Asparaginases (M5WUV5 and M5X0K4) are opposite to that of the amino acid (Figure 7). Thus, increased synthesis and decreased catabolism of Asn may conduct to an increase in this amino acid during very early development of peach fruit. In addition, the lack of detection of any asparagine synthase within the proteome (Supplementary Table 2), also support the hypothesis that the β-CAS-β-Cyano-Alanine hydratase could be a pathway for Asn synthesis in “Dixiland” peach fruit.

β-CAS is a key enzyme in cyanide detoxification (Blumenthal et al., 1968) and also the first step toward the synthesis of L-Asn in many species in the reaction catalyzed by the β-Cyano-Alanine hydratase (Castric et al., 1972; Machingura et al., 2016). The activity of these enzymes are present in fruit and flowers and increases during maturing process (Machingura et al., 2016). Hu et al. (2011) detected β-CAS in peach between 28 and 59 DAF. In agreement with our results, the levels of the protein increased in mesocarp during the S1–S2 transition. In endocarp, they detected a decrease from S1 to S2, while we sensed that decreased in the E to S1 transition. Asn together with Ala/Tyr, Asp, Gln, Glu, and γ-amino-butyrate are the amino acids transported at higher concentration by Prunus phloem (Douglas, 1993). Taken together, Asn synthesized in both mesocarp and endocarp could contribute to the Asn pool, which is also fed by import from the phloem. Further biochemical characterization of the enzymes of the β-CAS pathway in peach fruit is needed to reveal the importance of this pathway for fruit development. Considering the results presented here, together with previous work (Lombardo et al., 2011), we propose that Asn accumulated during very early peach development is further metabolized by Asparaginase during late development and ripening to provide skeletons for organic acids accumulation in the mesocarp.

### Photosynthetic Machinery Decays During Early Development in Both Mesocarp and Endocarp

It is generally accepted that sink organs as fruit and root rely on photosynthetic organs (mainly leaves) to growth and develop (Cocaliadis et al., 2014). Sugars and sugar alcohols such as sucrose and sorbitol are the main photosynthates imported to peach fruit from the phloem (Moing et al., 1997; Lombardo et al., 2011) which are further metabolized to render hexoses. In peach, fructose, glucose, sorbitol and sucrose increase as the fruit develops, mainly after S3 (Lombardo et al., 2011). In agreement, the activity of invertases and sorbitol dehydrogenase also increase over the development of peach fruit (Lombardo et al., 2011).

The occurrence of photosynthesis in fruit has been largely explored in tomato, including different aspects such as chloroplast to chromoplast conversion, the regulation of the expression of the photosynthetic components and the importance of photosynthesis during very early development (reviewed in Cocaliadis et al., 2014). The contribution of photosynthesis to total carbon of tomato fruit has been estimated to be up to 20% (Hetherington et al., 1998), but argued by others (Carrara et al., 2001). In comparison, our knowledge on the occurrence of photosynthesis in peach fruit is null. Proteins involved in light-harvesting complexes, electron transfer, Calvin cycle, photorespiration reactions and chlorophyll synthesis have been detected in peach fruit here by a massive proteomic approach (Supplementary Tables 2, 3). Chlorophylls have been detected as well (Figure 1D). On one hand, the higher levels of these proteins and chlorophylls in the mesocarp compared with the endocarp (Supplementary Figure 3 and Figure 1D) are in agreement with the outer location of the mesocarp and thus, its closer proximity to the light, suggesting that the system would probably be operating at least in the light capture phase. In addition, the presence of abundant starch grains in mesocarp (Supplementary Figure 9), although not necessary indicates that the carbon derives from carbon fixation, it shows enough carbon resources to be stored. During the transition from E to S1, and in concert with the fall in chlorophylls in the mesocarp, there is a decrease in the PSII light harvesting subunits, in Rubisco Small subunit and in other Calvin cycle enzymes (Supplementary Table 3). Nevertheless, abundant starch granules are still observed (Supplementary Figure 9). Further, in the transition from S1 to S2, an increase in the PSII light harvesting and polypeptide subunits, a decrease in key Calvin Cycle enzymes (sedoheptulose-bisphosphatase and phosphoglycerate kinase) and in starch, together with an increase in 6-phosphogluconate dehydrogenase oxidative pentose pathway, denote a demand on reductive power at stage S2 rather than on carbon fixation. In this line, at stage S2 the fruit almost does not increase in size. Taken together, these results suggest that mesocarp photosynthesis is possible to occur very early on the development, first providing both carbon and reductive power and latter only reductive power.

In the endocarp, the panorama is less clear at E and S1 as there are lower levels of chlorophyll and of proteins involved in photosynthesis compared with the mesocarp, in addition to the internal location in the pericarp (Figure 1D and Supplementary Table 3). In the transition from S1 to S2, PSII LHC and polypeptides, electron carriers, Rubisco small subunit and other Calvin enzymes decrease (Supplementary Table 3). Considering that at S2 the endocarp starts the lignification process it is highly probable that photosynthesis has no role at all. However, in tomato, it has been pointed out that fruit photosynthesis is critical for accurately timed seed development (Lytovchenko et al., 2011). Similarly, in endocarp of peach fruit photosynthesis may have a role in the seed development.

### Cell Wall Modifications Over the Early Development of Peach Fruit: Identification of Key Proteins Involved

The cell wall is an essential plant structure involved in numerous important developmental processes, like growth and cell division and fruit ripening (Cosgrove, 2005). Several studies have been undertaken to elucidate the cell wall changes during peach fruit ripening and softening (Brummell et al., 2004) and on how alterations in the cell wall structure affect the shelf life (Brummell and Harpster, 2001). It is widely documented that a solubilization or depolymerization of pectin and matrix glycans of the cell wall by the action of exo- and endo-polygalacturonases, endo-β-1,4-mannanase, α-L-arabinofuranosidase and β-galactosidase goes with the process of softening (Callahan et al., 1992; Trainotti et al., 2003; Brummell et al., 2004; Bustamante et al., 2012; Genero et al., 2016). On the contrary, a comprehensive research of the cell wall biosynthetic enzymes and proteins occurrence during peach development is still missing.

During very early peach development, the fruit undergo a burst of cell division and elongation at E and S1, with a pause in S2, as accounted by modifications in the fruit volume, cell size and cell wall width and cellulose and aniline blue staining (Figure 8, 9 and Supplementary Figures 1B, 6). In addition, a decrease in the level of proteins involved in cell division is detected in endocarp and mesocarp at S2 (ppa005822m, ppa009766m, ppa015773m; Supplementary Table 3). Cell expansion involves modifications in cell wall structure. The increases in XETs, FLAs and leucine-rich repeat family proteins/extensins (LRX) in the endocarp and in the mesocarp in the transition from E to S1 (Table 1) are in agreement with modifications in cell wall. Through cell enlargement and elongation, the structure of the cell wall is relaxed and then strengthened. In peach, a decrease in cellulose deposition is observed at S1, in agreement with an increase in cellulase activity at this stage described earlier by Bonghi et al. (1998). XETs modulate cell wall strength, flexibility and porosity, and cell expansion by linking xyloglucans with cellulose, and xyloglucans with (1,3; 1,4)-β-D-glucans (Eckardt, 2004; Nishikubo et al., 2011). LRXs are also cell wall-localized proteins involved in the regulation of plant growth (Draeger et al., 2015). In addition, FLAs are a subfamily of arabinogalactan proteins that participate in cell expansion and adhesion (Johnson et al., 2003). Thus, in “Dixiland” peach fruit, these cell wall proteins might have a key participation in the fast growth of the very early stage as reported for XETs and LRX in watermelon (Guo et al., 2011). Other proteins involved in the synthesis of cell wall precursors were also identified as highly induced in the transition from E to S1 in the endocarp (Table 1 and Supplementary Table 3), like mannose-1-phosphate guanylyltransferase that provides GDP-mannose that is used to add mannose residues to cell wall molecules and as well as a precursor of GDP-fucose for the addition of fucose residues in the cell wall (Lukowitz et al., 2001). Later, in the transition from S1 to S2, and in agreement with the cease in growth of peach fruit at S2, a decrease in the levels of many isoforms of Polygalacturonase and Beta-D-xylosidase, key enzymes during fruit ripening, in both endocarp and mesocarp is observed (Table 1). In agreement, the decrease in the transcript encoding a Beta-D-xylosidase was observed in the transition from S1 to S2 in peach fruit (Di Santo et al., 2009).

### Proteins Involved in Signaling and RNA Metabolism Vary Spatial and Temporally During Very Early of Peach Development

Processes of cell division and expansion, and tissue differentiation require tight regulation both at the level of gene activity and translation. These events are, in addition, coupled to phytohormone levels. Changes in the levels of auxins, gibberellins and cytokinins are key signals during early fruit development (Bonghi et al., 2011). Here, we have shown that changes in protein metabolism are key to fruit development. In addition, as it is show in Figure 2, 5, the functional categories signaling and RNA metabolism are well represented among proteins changing across development in both mesocarp and endocarp, and also between endocarp and mesocarp. In consequence, it is not surprising the observation of variable proteins involved in RNA processing, RNA binding, regulation of transcription, calcium signaling, or participating in the signaling mediated by protein G, receptor kinases, phosphoinositides, MAP kinases and 14-3-3 proteins (Supplementary Table 3) during fruit development. Future studies could get more insight into these responses.

## Conclusion

Fruit yield relies on a set of developmental processes, which include flower initiation and differentiation, fertilization, fruit set and development (Hanke et al., 2007). Each aspect may limit fruit production. Peach is an important fruit crop and have been turned into a valuable model, together with tomato, for the research of climacteric fruits. Here we got insight into the early stages of fruit development with distinction of the events in endocarp and mesocarp. In this respect, we provide valuable information on the nature and abundance of the proteins present in these tissues very early after pollination. This information, coupled with profiles of metabolites and transcripts available, provides novel insights into the biology of peach fruit development preceding pit hardening.

## Author Contributions

CR, CAB, COB, and GM conducted the experiments. CAB, MD, and ML conceived the project. MD and ML wrote the manuscript.

## Conflict of Interest Statement

The authors declare that the research was conducted in the absence of any commercial or financial relationships that could be construed as a potential conflict of interest.

## Footnotes

1 http://primer3.ut.ee/

2 http://mev.tm4.org

3 http://www.perseus-framework.org

4 http://www.tm4.org/

5 https://phytozome.jgi.doe.gov/pz/portal.html
