# Supplementary material for: Peach Fruit Development: A Comparative Proteomic Study Between Endocarp and Mesocarp at Very Early Stages Underpins the Main Differential Biochemical Processes Between These Tissues
Source: Front Plant Sci. 2019 Jun 4;10:715. doi: 10.3389/fpls.2019.00715 (PMC6558166; doi:10.3389/fpls.2019.00715)

Supplementary Figure 1. (A) “Dixiland” peach growth curve. Fruits were collected at different days after flowering (DAF). A typical peach curve is observed, where light blue corresponds to E stage, blue to the S1 phase, black to S2 stage, green to the S3 period and red to the final phase S4. DAF pointed out with an arrow were considered as representative of E, S1 and S2, and used for further experiments. (B) Fruit volume of representative fruit of E, S1 and S2.

A

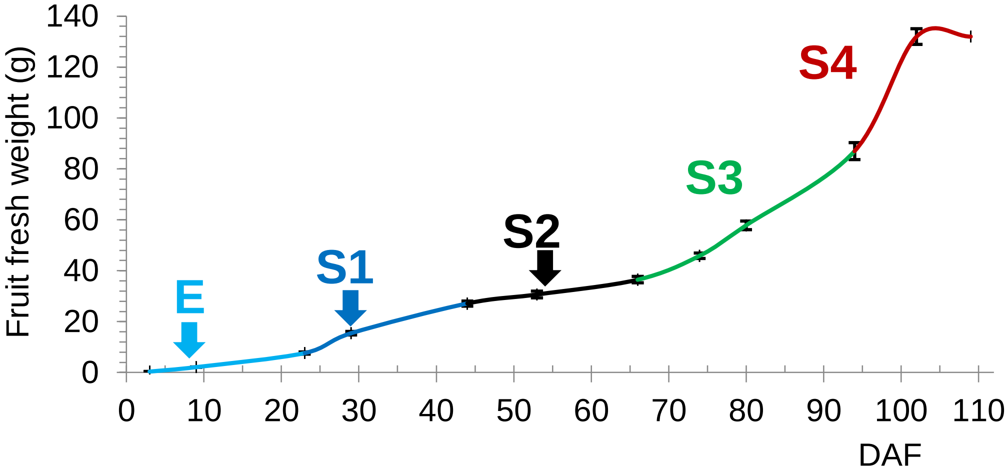

B

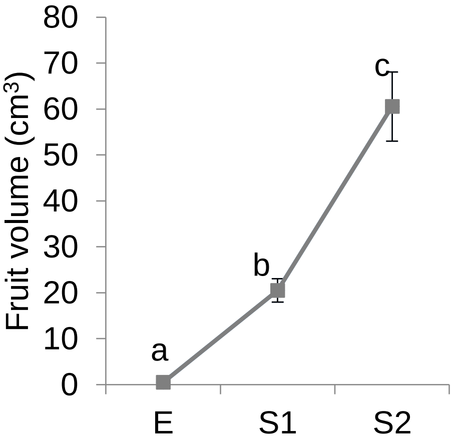

Supplement: Supplementary file 1 [file Data_Sheet_1.PDF]
