# Supplementary material for: Peach Fruit Development: A Comparative Proteomic Study Between Endocarp and Mesocarp at Very Early Stages Underpins the Main Differential Biochemical Processes Between These Tissues
Source: Front Plant Sci. 2019 Jun 4;10:715. doi: 10.3389/fpls.2019.00715 (PMC6558166; doi:10.3389/fpls.2019.00715)

Supplementary Figure 2. Plots of the three first components of principal component analysis of protein occurrence and abundance during very early peach fruit development. PCA was conducted using Clustvis (Metsalu and Vilo, 2015). Proteomes of peach fruit mesocarp (m) and endocarp (e) were analyzed at E, S1 and S2 developmental stages. (A) Principal component 2 (PC2) versus principal component 1 (PC1) showing the 53.2 % of the variation. (B) Principal component 3 (PC3) versus PC1 explaining the 51.0 % of the variations. (C) PC3 vs PC2 accounting a 42.0 % of the protein variation.

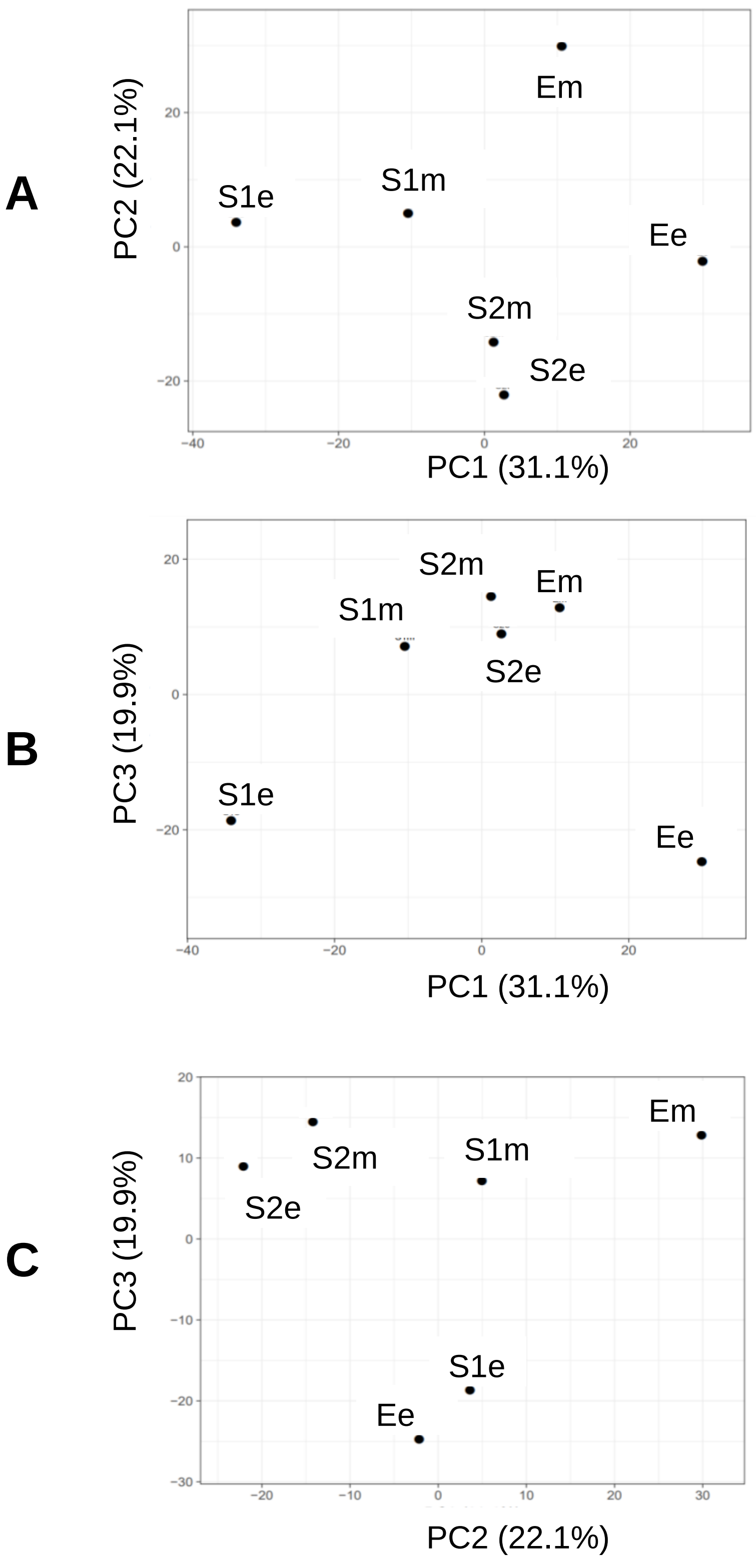

Supplement: Supplementary file 2 [file Data_Sheet_2.PDF]
