# Supplementary material for: Peach Fruit Development: A Comparative Proteomic Study Between Endocarp and Mesocarp at Very Early Stages Underpins the Main Differential Biochemical Processes Between These Tissues
Source: Front Plant Sci. 2019 Jun 4;10:715. doi: 10.3389/fpls.2019.00715 (PMC6558166; doi:10.3389/fpls.2019.00715)

Supplementary Figure 3. Analysis of photosynthetic related proteins that exhibit a different abundance in endocarp and mesocarp. Green and magenta boxes represent the proteins increased and decreased, respectively, in peach mesocarp with respect to endocarp at E, S1 or S2 developmental stages. Values are expressed in log2 bases. Grey boxes represent that no significant variation between tissues was detected.

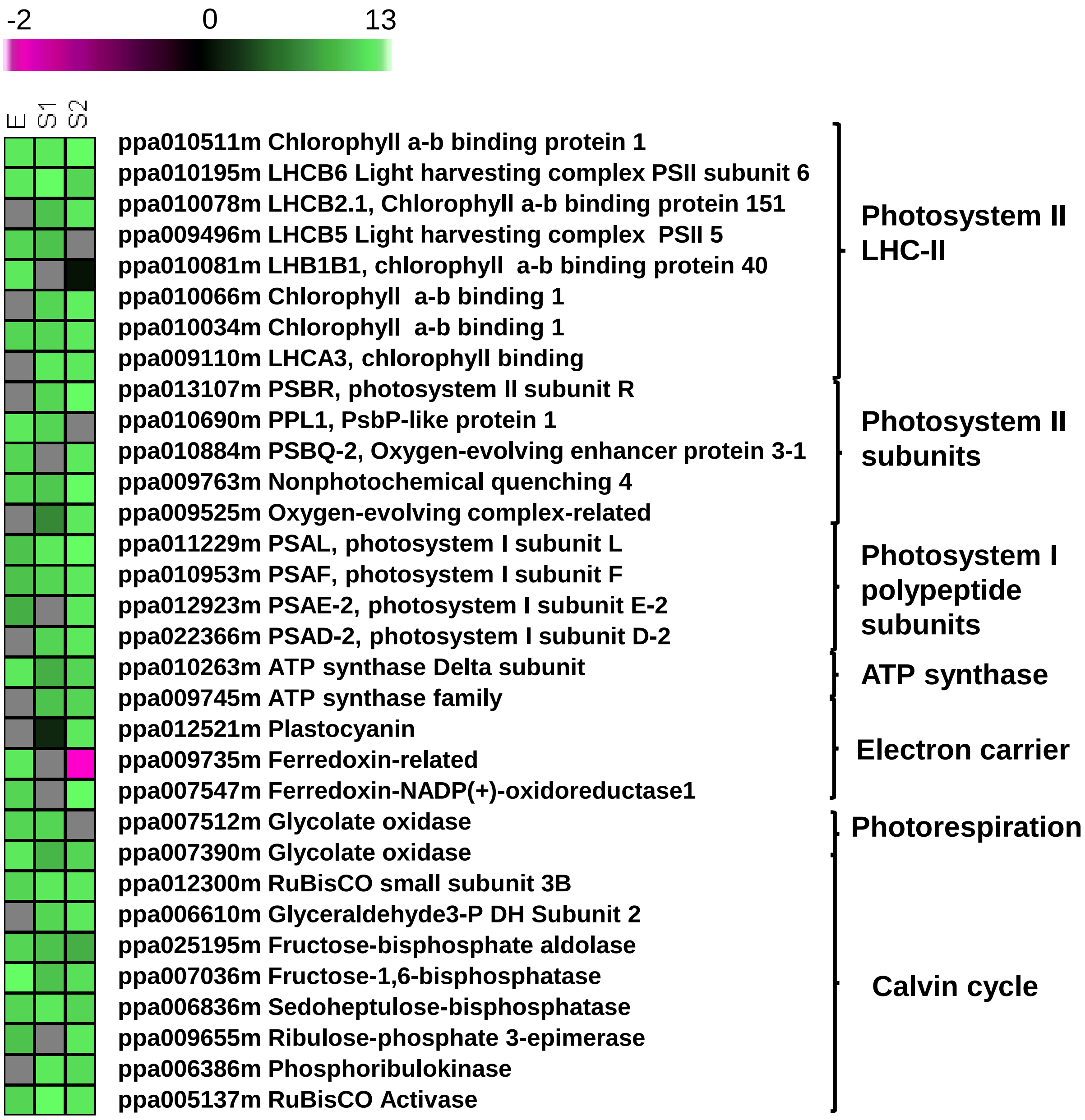

Supplement: Supplementary file 3 [file Data_Sheet_3.PDF]
