# Supplementary material for: Peach Fruit Development: A Comparative Proteomic Study Between Endocarp and Mesocarp at Very Early Stages Underpins the Main Differential Biochemical Processes Between These Tissues
Source: Front Plant Sci. 2019 Jun 4;10:715. doi: 10.3389/fpls.2019.00715 (PMC6558166; doi:10.3389/fpls.2019.00715)

Supplementary Figure 4. Overview of variable proteins at stages E, S1 and S2 based on their correspondent metabolic pathways. Each square corresponds to a protein. Blue and red indicate lower and higher expression in mesocarp than in endocarp, respectively, in a log2 basis. Scale bar is at the top right of each figure. Images were generated using MapMan program (Usadel et al., 2009).

E

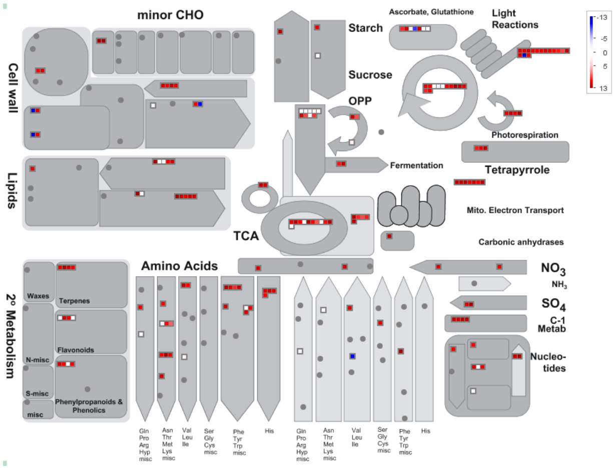

S1

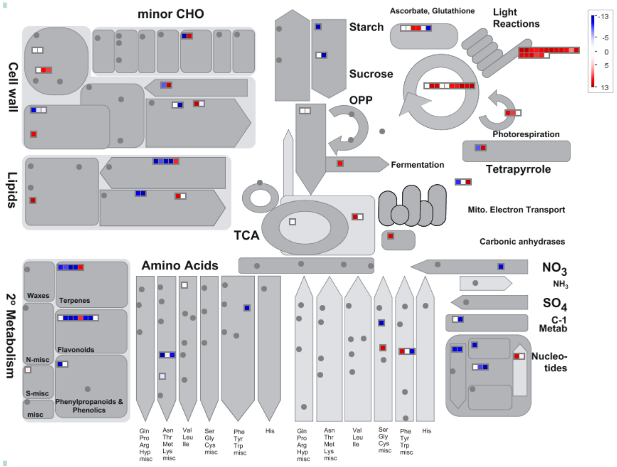

S2

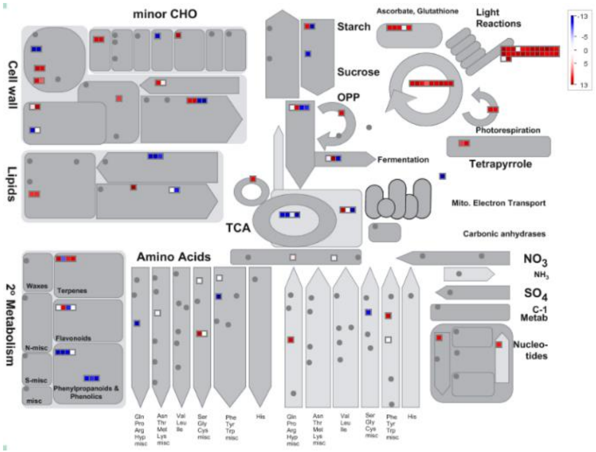

Supplement: Supplementary file 4 [file Data_Sheet_4.PDF]
