# Supplementary material for: Peach Fruit Development: A Comparative Proteomic Study Between Endocarp and Mesocarp at Very Early Stages Underpins the Main Differential Biochemical Processes Between These Tissues
Source: Front Plant Sci. 2019 Jun 4;10:715. doi: 10.3389/fpls.2019.00715 (PMC6558166; doi:10.3389/fpls.2019.00715)

Supplementary Figure 5. Principal component analysis (PCA) of the amino acid profile obtained by HPLC. The variance explained by each component (%) is indicated in parentheses.

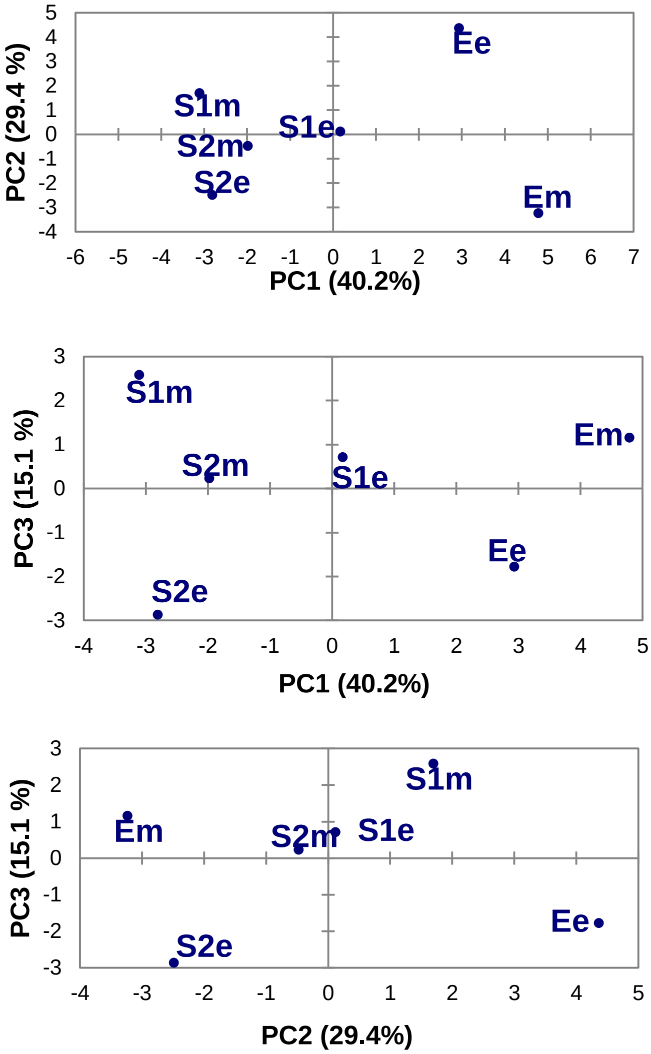

Supplement: Supplementary file 5 [file Data_Sheet_5.PDF]
