# Supplementary material for: Peach Fruit Development: A Comparative Proteomic Study Between Endocarp and Mesocarp at Very Early Stages Underpins the Main Differential Biochemical Processes Between These Tissues
Source: Front Plant Sci. 2019 Jun 4;10:715. doi: 10.3389/fpls.2019.00715 (PMC6558166; doi:10.3389/fpls.2019.00715)

Supplementary Figure 6. Cell count in an area basis as a measurement of cell sizes. The number of cells per images of 51,042 µm2 collected with 60X magnification was counted at each stage and tissue and shown. Within each tissue, values with different letters are statically significant different ($p < 0.05$).

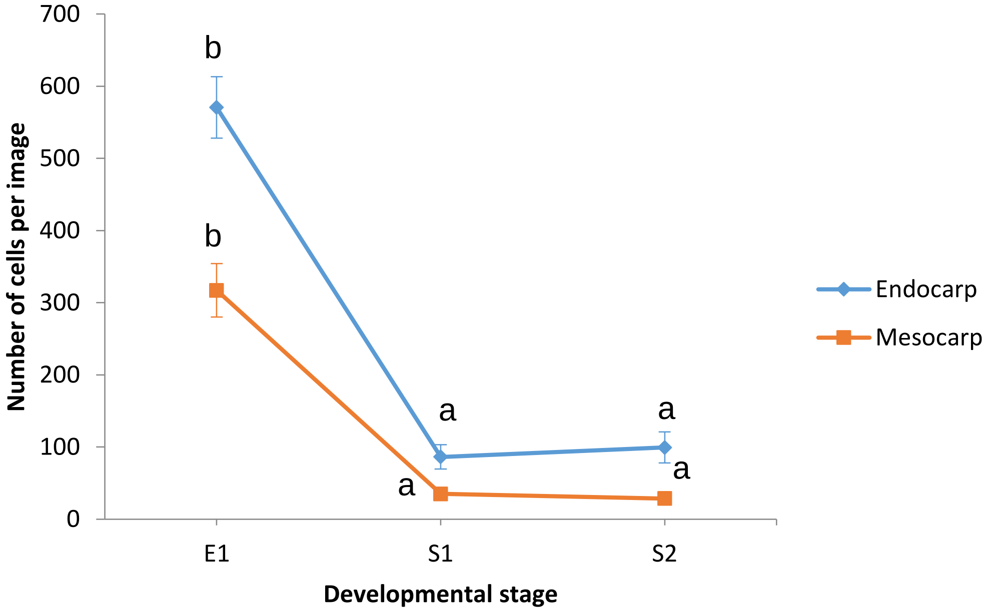

Supplement: Supplementary file 6 [file Data_Sheet_6.PDF]
