# Supplementary material for: Peach Fruit Development: A Comparative Proteomic Study Between Endocarp and Mesocarp at Very Early Stages Underpins the Main Differential Biochemical Processes Between These Tissues
Source: Front Plant Sci. 2019 Jun 4;10:715. doi: 10.3389/fpls.2019.00715 (PMC6558166; doi:10.3389/fpls.2019.00715)

Supplementary Figure 7. Aniline Blue staining of endocarpic (A) and mesocarpic (B) cells at E, S1 and S2. Magnification used: 60X. Scale bars denote 25 μm. Red and blue channels were integrated in each image.

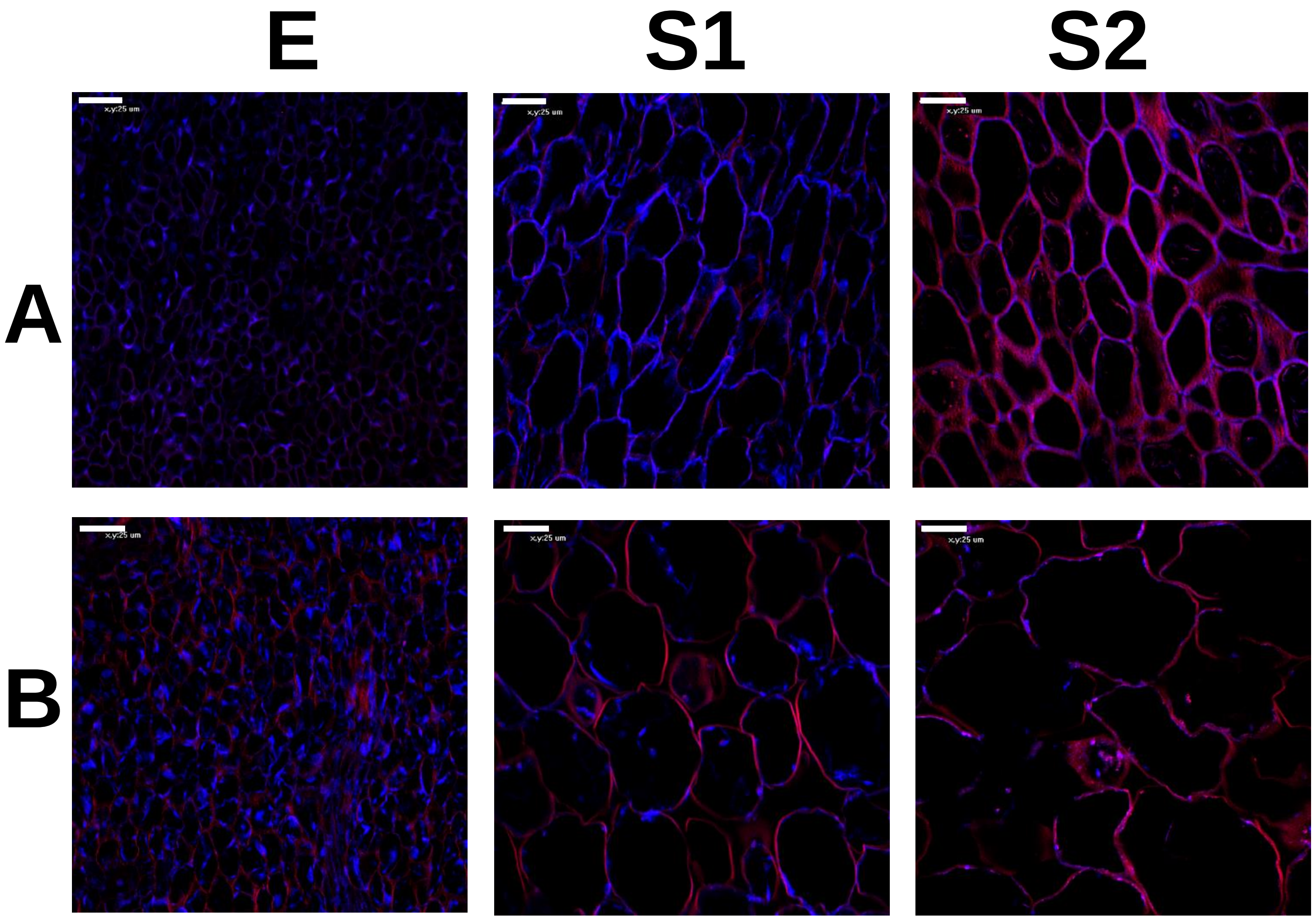

Supplement: Supplementary file 7 [file Data_Sheet_7.PDF]
