# Supplementary material for: Peach Fruit Development: A Comparative Proteomic Study Between Endocarp and Mesocarp at Very Early Stages Underpins the Main Differential Biochemical Processes Between These Tissues
Source: Front Plant Sci. 2019 Jun 4;10:715. doi: 10.3389/fpls.2019.00715 (PMC6558166; doi:10.3389/fpls.2019.00715)

Supplementary Figure 9. PAS staining for starch grains. Light micrographs showing cross sections of endocarp (A and B) and mesocarp (C and D) collected at E, S1 and S2 are shown. Arrowheads indicate some of the starch granules. The size of the scale bars is indicated in each figure. Magnification used: 40 X (A and C) and 100 X (B and D).

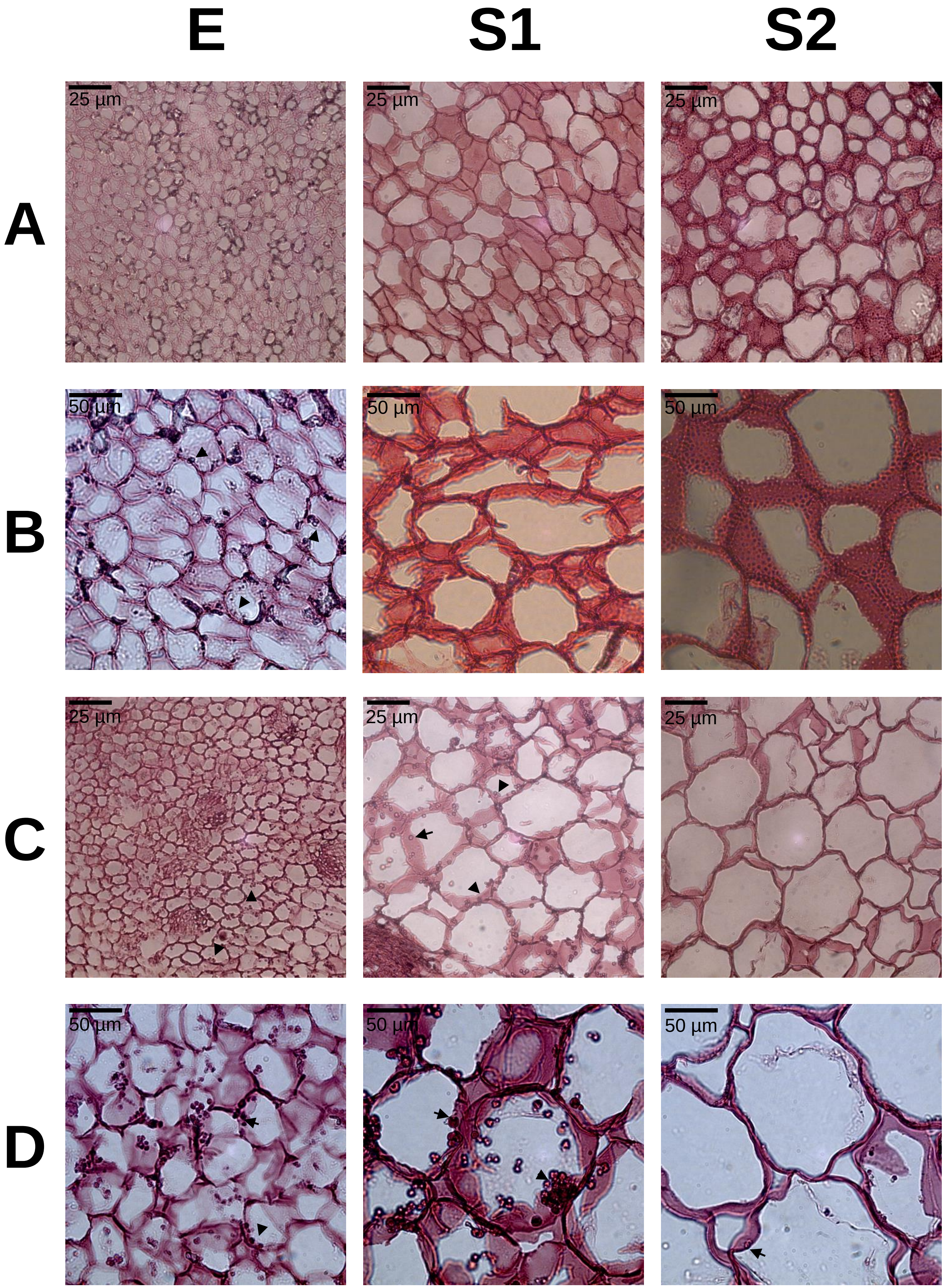

Supplement: Supplementary file 9 [file Data_Sheet_9.PDF]
